# Investigation of the Effect of B_4_C Addition on the Matrix Composition of Diamond Sockets in Granite Cutting and the Relationships Between Socket Metallographic Properties and Cuttability Parameters via SEM and Regression Analysis

**DOI:** 10.3390/ma19194079

**Published:** 2026-09-24

**Authors:** Gökhan Ekincioğlu, Raşit Altındağ

**Affiliations:** 1 Kaman Vocational School, Department of Mining and Mineral Extraction, Ahi Evran University, Kırşehir 40100, Türkiye; 2 Department of Mining Engineering, Faculty of Engineering, Süleyman Demirel University, Isparta 32260, Türkiye; rasitaltindag@sdu.edu.tr

**Keywords:** natural stone, diamond socket, metallographic properties, boron carbide, specific cutting energy, specific wear, SEM analysis

## Abstract

Selecting diamond sockets with matrix properties compatible with the target rock is critical for cutting efficiency and cost reduction in natural stone processing. This study investigated the effect of B_4_C addition (0–7 wt.%) on the matrix microstructure, diamond–matrix interfacial bonding quality, and cutting performance of powder metallurgy diamond sockets used in granite sawing. Eight sockets, one undoped and seven with varying B_4_C contents, were sintered at 730 °C, 35 MPa, for 3 min. Unit volume weight (UVW), porosity (P), Knoop hardness (HK), and abrasion loss by weight (ALW) were determined for each composition. SEM analyses on fracture surfaces revealed significant microstructural differences with increasing B_4_C content. Pore sizes increased from 2–4 µm (0% B_4_C) to over 40 µm (7% B_4_C). The 4% B_4_C socket achieved the most uniform B_4_C grain distribution (3–8 µm), the highest Knoop hardness (303.46 HK), the lowest porosity (4.20%) among doped sockets, and the tightest diamond–matrix bonding. Cutting tests on Hisar Grey granite confirmed that the 4% B_4_C socket delivered the highest net power consumption (720.85 W) and the lowest specific wear (84.84 µc/m^2^), consistent with SEM observations. Regression analyses identified porosity as the single strongest predictor across all cutting parameters (r = 0.87–0.97), and abrasion loss by weight as a reliable indicator of specific wear (r = 0.98). These findings suggest that laboratory metallographic measurements may serve as useful indicators of cutting performance trends, though validation across different rock types and cutting conditions is needed before broader generalisations can be drawn.

## 1. Introduction

Natural stone cutting is performed using cutting tools known as sockets, which consist of two main components: diamond and matrix. Selecting sockets with properties compatible with the engineering characteristics of the natural stone to be cut is of critical importance for improving cutting efficiency and reducing energy consumption and tool costs.

During the processing of natural stone, frequently encountered problems include short tool life and inefficient cutting processes resulting from irregular wear of the matrix and diamond grains. These issues adversely affect both cutting precision and the overall production costs of natural stone. Therefore, the development of diamond sockets appropriate to the structural properties of the rock to be cut, along with the determination of optimal cutting conditions, is essential for achieving high cutting efficiency.

A number of researchers have investigated the development of socket matrices, examining production parameters such as sintering time, temperature, and pressure, alongside diamond content variations and the effects of elements including iron, cobalt, titanium, molybdenum, copper, and nickel on matrix properties [1,2,3,4,5,6,7,8,9,10,11]. Moreover, B_4_C has been proposed as a partial substitute for cobalt in powder metallurgy diamond tool matrices, offering cost reduction potential alongside its well-known hardening effect [2,4,5]. The powder metallurgy method, which is widely used in diamond socket production, has been shown to play a decisive role in shaping the microstructural properties and mechanical performance of sockets [12,13]. Production parameters and material selection in particular have a direct bearing on tool wear behaviour and cutting efficiency [12]. That said, most of these studies have stayed within the bounds of metallographic investigation. In a more recent contribution, Li et al. [14] used SEM microstructural analyses and microhardness measurements to assess the brazing joint quality of granite and marble diamond segments, finding that porous surfaces produced by powder metallurgy considerably accelerate diffusion at interfaces—with direct consequences for bond strength and cutting performance.

A review of the scientific literature on rock cuttability reveals that studies have generally examined the relationships between mineralogical, physical, and mechanical properties of rocks and cuttability parameters such as cutting forces, specific cutting energy, specific wear, and noise level obtained under varying operational conditions [15,16,17,18,19,20,21,22,23,24,25,26,27,28,29,30,31,32,33,34,35].

In the context of diamond saw applications for natural stone cutting, the relationship between tool microstructure and cutting performance has attracted growing scientific interest [36]. The wear behaviour of diamond tools with different matrix compositions—including parameters such as specific cutting energy and wear rate—has been investigated in stone cutting operations [37,38]. Nevertheless, the relationships between the metallographic properties induced by additives and cuttability parameters remain insufficiently understood.

A review of the existing literature indicates that studies specifically examining the influence of metallographic properties of diamond sockets on cuttability parameters are notably limited. Ekincioğlu and Altındağ [39] investigated the effect of boron carbide addition on circular saw cutting performance, evaluating the performance criteria of B_4_C-doped and undoped diamond sockets in terms of cutting force, wear resistance, and specific cutting energy, and identifying the optimum B_4_C content. However, that study did not address the microstructural mechanisms underlying the relationships between metallographic properties and cuttability parameters. In a related study conducted using the same socket set, Ekincioğlu et al. [40] examined the correlation between ultrasonic velocity and metallographic and cutting performance parameters.

The present study focuses on the relationships between the metallographic changes induced by B_4_C addition and the cuttability parameters of granite, rather than on the direct effect of boron carbide-doped diamond sockets produced by powder metallurgy on cutting parameters. In the socket matrix composition used in this study, B_4_C was introduced as a one-to-one replacement for cobalt. Accordingly, the observed changes in the metallographic properties of the sockets reflect the combined effect of increasing B_4_C content and decreasing cobalt content. The focus of this study is therefore not to isolate the individual contribution of either constituent, but to reveal the effects of the metallographic properties arising from this substitution on cuttability parameters. Although Ekincioğlu and Altındağ [39,40] previously examined the cutting performance of B_4_C-doped sockets, the microstructural mechanisms underlying the relationships between metallographic properties and cuttability parameters were not addressed in those studies. To the best of the authors’ knowledge, no study has employed regression analyses of metallographic properties and cuttability parameters of B_4_C-doped diamond sockets in natural stone cutting in conjunction with SEM-based microstructural investigation. The present study aimed to highlight the significance of the relationship between two distinct disciplines—powder metallurgy and natural stone cutting.

The experimental data presented in this study (including the metallographic measurements of the sockets and the cutting performance parameters) were originally obtained within the scope of the first author’s PhD thesis [41]. The SEM images were likewise obtained in that context. The present manuscript presents, for the first time, a systematic regression-based analysis linking metallographic properties to cuttability parameters, supported by SEM-based microstructural interpretations that were not developed in the thesis or in the previous publications [39,40].

## 2. Materials and Methods

Carbides are incorporated into the matrix to enhance the wear resistance of the metallic matrix and to strengthen the bond at the diamond–matrix interface. Boron carbide (B_4_C), characterised by its high melting point, high hardness, low density, and excellent mechanical properties, is an important material for advanced technological applications and is widely used to improve wear resistance [5,39,42,43].

Within the scope of this study, eight diamond sockets were produced by the powder metallurgy method—one undoped (0% B_4_C) and seven with varying B_4_C contents (1%, 2%, 3%, 4%, 5%, 6%, and 7%). The sockets were sintered under industrial conditions at a pressure of 35 MPa, a temperature of 730 °C, and a sintering duration of 3 min. In the socket matrix composition, the proportions of nickel, bronze, and diamond were kept constant, while the cobalt content was progressively reduced with increasing B_4_C addition (Table 1). The B_4_C powder used in this study was supplied by BD Chemistry and Chemical Materials Trading Ltd. Co. (Kayseri, Türkiye).

The sockets were subsequently welded onto 350 mm-diameter steel discs to produce eight circular saws suitable for granite cutting. In the experimental programme, the metallographic properties of the diamond sockets—namely unit volume weight (UVW), porosity (P), Knoop hardness (HK), and abrasion loss by weight (ALW)—were first determined. General views of the produced sockets and the circular saws are presented in Figure 1.

Following the determination of metallographic properties, Scanning Electron Microscopy (SEM) analyses were performed on the fracture surfaces of both undoped and B_4_C-doped sockets at varying addition ratios using a LEO 1430 VP scanning electron microscope (LEO Electron Microscopy Ltd., Cambridge, UK), located at the Technology Application and Research Center of Afyon Kocatepe University, Afyonkarahisar, Turkiye. These analyses were conducted to investigate the effect of B_4_C addition on the socket matrix microstructure and to elucidate the microstructural mechanisms underlying the regression relationships between metallographic properties and cuttability parameters identified in the Relationships Between Metallographic Properties and Cutting Parameters Section. The SEM analyses focused on pore size distribution, B_4_C particle dispersion homogeneity, and diamond–matrix interfacial bonding quality within the socket matrix.

Cutting experiments were carried out on Hisar Gray, a quartz monzonite with a uniaxial compressive strength of 206.63 MPa, a Knoop hardness of 445.82 HK, and a SiO_2_ content of 67.82%, using specimens of 5 cm × 10 cm × 25 cm dimensions with a homogeneous structure. The high SiO_2_ content, compressive strength, and hardness of Hisar Gray indicate that it is a hard and abrasive rock type. The fact that the Knoop hardness of the rock (445.82 HK) is considerably higher than the maximum socket hardness value (303.46 HK—4% B_4_C) places significant demand on the socket matrix during cutting and renders the metallographic properties of the socket particularly critical for cutting efficiency. Operational parameters—including peripheral speed, feed rate, and depth of cut—were kept constant throughout the cutting tests (Table 2).

The cutting experiments were conducted using the test apparatus located in the Natural Stone Technology Laboratory of the Department of Mining Engineering, Süleyman Demirel University (Isparta, Turkey). Figure 2 illustrates the general view of the cutting test set-up and a view captured during the cutting operation.

The appearance of the block specimens following the cutting tests is presented in Figure 3. After completion of the cutting experiments, the net power consumption, specific cutting energy, and specific wear values of the circular saws were determined. The data obtained from the experimental programme are presented in the Findings Section.

Simple regression analyses were applied to quantify the relationships between the metallographic properties of B_4_C-doped diamond sockets and cuttability parameters. All statistical analyses were performed using Microsoft Excel 2019 (Microsoft Corporation, Redmond, WA, USA) (Data Analysis ToolPak) and IBM SPSS Statistics v26 (IBM Corporation, Armonk, NY, USA). For the Pnet–HK, SCE–HK, and SW–HK variable pairs, the undoped socket (0% B_4_C) was excluded from the regression analysis as a sensitivity test, in order to separately evaluate the hardness–cutting performance relationship among the B_4_C-doped sockets, which differ in matrix composition from the undoped socket. This exclusion was performed post hoc, and both analyses (n = 8 and n = 7) are reported together in the interest of transparency.

For each composition, three independently manufactured sockets were used for Knoop hardness and porosity measurements, and two independently manufactured sockets were used for weight-loss wear testing. All results are reported as mean ± standard deviation. Cutting performance parameters (net power consumption and specific cutting energy) were obtained from a single saw blade per composition over 60 consecutive cuts; the reported values represent the mean of these 60 measurements, and the associated standard deviation reflects within-test measurement variability rather than between-specimen variability. This distinction is acknowledged as a limitation of the cutting test protocol.

## 3. Results

### 3.1. Metallographic Properties of the Sockets

UVW values were measured in accordance with the ASTM B962 standard [44], which was specifically developed for compacted or sintered powder metallurgy products possessing interconnected porosity. The samples were first weighed in air (A), then immersed in pure water using a tared metal basket and weighed again (B). UVW was calculated as follows (Equation (1)):

UVW = (A × dw)/(A − B)(1)

where dw is the density of water (g/cm^3^). Subsequently, for each composition, relative density (RD) and porosity (P) were determined using Equations (2) and (3), based on the ratio of the measured UVW value to the theoretical density (TD) calculated from the powder mixture proportions:

RD (%) = (UVW/TD) × 100(2)

P (%) = 100 − RD(3)

It was observed that the UVW value decreased as the B_4_C content increased; this is attributed to the fact that the density of boron carbide (2.52 g/cm^3^) is lower than the densities of the matrix components—cobalt (8.90 g/cm^3^), nickel (8.85 g/cm^3^), and bronze (8.60 g/cm^3^) [5,45].

The disparity between the melting points of boron carbide and the metallic matrix materials promoted void formation within the socket structure. A particularly pronounced increase in porosity (P) was observed at B_4_C contents exceeding 5%. The microstructural origins of this porosity increase were further investigated through SEM analysis, as presented in Scanning Electron Microscopy (SEM) Analyses of the Sockets Section.

Knoop hardness (HK) values were determined by taking 50 measurements from the surface of each socket at regular intervals, yielding a total of 1200 measurements, which were subsequently averaged. The maximum HK value was recorded at the 4% B_4_C addition ratio. Assessment of ALW values confirmed that the 4% B_4_C-doped socket exhibited the highest wear resistance among all compositions tested. The metallographic measurement results are summarised in Table 3.

### 3.2. Scanning Electron Microscopy (SEM) Analyses of the Sockets

Scanning Electron Microscopy (SEM) analyses were performed on the fracture surfaces of both undoped and B_4_C-doped sockets at varying addition ratios, with the aim of investigating the effect of boron carbide on the socket matrix microstructure and elucidating the microstructural mechanisms underlying the regression relationships identified in the Relationships Between Metallographic Properties and Cutting Parameters Section. The SEM analyses provide qualitative microstructural observations with respect to pore size distribution, B_4_C particle dispersion homogeneity, and diamond–matrix interfacial bonding quality. The SEM analyses were conducted qualitatively; quantitative image analysis, pore-size statistics, and compositional verification by EDS were not performed. Accordingly, the microstructural interpretations presented are based on visual observations and should be regarded as indicative rather than definitive.

In the undoped socket (0% B_4_C), a homogeneous morphology was observed. Consistent with the low porosity value obtained from metallographic measurements, pores within the structure were limited in number and measured approximately 2–4 µm in size (Figure 4a). However, examination of the diamond–matrix interface revealed a gap of approximately 8 µm (Figure 4b), indicative of poor interfacial bonding. This deficiency is thought to stem from the inadequate wetting performance of the matrix material under the applied sintering conditions, thereby limiting its ability to form sufficient bonds with diamond grains. The resultant weak bonding is consistent with the comparatively high specific wear value recorded for the undoped socket.

In the 1% B_4_C-doped socket, an increase in porosity was observed, with pores ranging from approximately 10–12 µm in size (Figure 4c). Notably, the diamond–matrix interfacial bond was found to be improved relative to the undoped socket (Figure 4d). This enhancement in bonding quality (evident even at low B_4_C content) may partly account for the reduction in specific cutting energy observed at the 1% B_4_C addition ratio.

In the 2% B_4_C-doped socket, porosity continued to increase; however, the matrix structure was found to be more regular compared to the 1% B_4_C socket (Figure 4e). This regularisation of the matrix structure is considered to contribute to the observed increase in microhardness. Diamond–matrix interfacial bonding was similarly found to be of good quality, comparable to that of the 1% B_4_C socket (Figure 4f). The improved matrix regularity at 2% B_4_C is consistent with the higher HK value (253.35) obtained from metallographic measurements.

In the 3% B_4_C-doped socket, cracks of approximately 40–50 µm were observed within the matrix (Figure 4g); nevertheless, a good diamond–matrix interfacial bond was identified (Figure 4h). The cracks observed at this addition level may be associated with localised thermal stresses arising from the melting point mismatch between boron carbide and the metallic matrix constituents.

In the 4% B_4_C-doped socket, B_4_C grains measuring 3–8 µm were observed (Figure 4i), and a more homogeneous distribution of B_4_C was identified compared to all other sockets. This homogeneous distribution is considered responsible for the maximum hardness (HK = 303.46) achieved at the 4% B_4_C ratio. Furthermore, the matrix was observed to fracture in a lamellar manner, and a regular diamond–matrix interface was identified (Figure 4j). The co-occurrence of homogeneous B_4_C distribution, high hardness, and low porosity (4.20%) at the 4% B_4_C addition ratio is consistent with the regression findings: the highest net power consumption (720.85 W) and lowest specific wear (84.84 µc/m^2^) were both recorded at this ratio, suggesting that harder and denser sockets require greater energy input while exhibiting reduced wear.

In the 5% B_4_C-doped socket, a non-homogeneous distribution of B_4_C was observed within the matrix (Figure 4k), accompanied by a coarse-grained structure with weakly bonded constituents (Figure 4l). This heterogeneous distribution is suggested to be attributable to the inadequacy of sintering conditions as the B_4_C content exceeds a threshold level. Optimisation of sintering parameters—including sintering time, pressure, and temperature—is necessary to achieve a more homogeneous matrix structure at higher B_4_C addition ratios. The deterioration in matrix quality observed at 5% B_4_C is consistent with the abrupt increase in porosity (8.70%) recorded in metallographic measurements.

In the 6% B_4_C-doped socket, pores of approximately 35–40 µm were identified within the matrix (Figure 4m,n). In the 7% B_4_C-doped socket, the progressive increase in porosity continued (Figure 4o,p), with the highest porosity values of 11.93% and 14.31% recorded for the 6% and 7% B_4_C sockets, respectively, in full agreement with metallographic measurements. The large pores observed in SEM images at high B_4_C contents provide a mechanistic explanation for the regression findings: as porosity increases, matrix wear accelerates, fresh diamond grains are continuously exposed at the socket surface, cutting is facilitated, and consequently net power consumption and specific cutting energy decrease while specific wear increases markedly. This observation is consistent with the strong negative exponential correlation (r = 0.87) identified between net power consumption and porosity in the regression analysis.

When these findings are evaluated as a whole, the observed microstructural changes stem from the simultaneous increase in the B_4_C content and decrease in the cobalt content within the socket. It is not possible to isolate the independent contribution of each variable within the framework of this composition design.

The increase in porosity and the decrease in density observed with rising B_4_C content can be attributed to sintering conditions. Co acts as a binder in the powder metallurgy diamond socket matrix. At the sintering temperature of 750 °C, it partially melts, filling the inter-particle voids and promoting the formation of a dense microstructure. B_4_C, by contrast, remains in the solid phase at relatively low sintering temperatures due to its considerably higher melting point (approximately 2450 °C). Consequently, the simultaneous decrease in Co content accompanying the increase in B_4_C content restricts liquid-phase formation and adversely affects sinterability. Indeed, a pronounced increase in porosity (P: from 8.70% to 14.31%) and a corresponding decrease in density were observed in compositions containing 5% B_4_C and above. At these addition levels, the weakening of Co’s binding effect led to heterogeneous particle distribution and coarse matrix grain formation, which in turn resulted in deterioration of interfacial bonding quality. This microstructural degradation directly affected not only the mechanical properties but also the wear behaviour during cutting.

### 3.3. Cuttability Parameters

#### 3.3.1. Specific Cutting Energy and Net Power Consumption

Specific cutting energy (SCE), expressed in J/mm^3^, refers to the energy required to remove a unit volume of rock. An efficient cutting process is achieved by maximising cutting rate while minimising specific cutting energy; under such conditions, the cutting operation becomes most economical, and SCE serves as a direct indicator of cutting efficiency [25].

Instantaneous power values were first recorded to calculate SCE, and a power–time graph was subsequently plotted (Figure 5). The graph illustrates power consumption during both unloaded operation and full cutting. The net power consumed for cutting is defined as the difference between these two values (Equation (4)):

(4) Pnet=Pfl−Pul

Pnet = net power *consumption* for cutting, watt.
Pfl = power value drawn when the saw is at full load, watt.
Pul = power value is drawn when the saw is unloaded, watt.

(5) $$SCE=\frac{Pnet}{0.1666}\times h\times Ws\times Vi$$

*SCE* = specific cutting energy, j/mm^3^.
*h* = depth of cut, mm.
*Ws* = socket width, mm.
*Vi* = feed rate, cm/min.

Analysis of the experimental data revealed that the highest SCE and net power consumption values were recorded for the 4% B_4_C-doped circular saw. As corroborated by SEM analysis (Scanning Electron Microscopy Analyses of the Sockets Section), the 4% B_4_C socket exhibited the most homogeneous B_4_C grain distribution (3–8 µm) and the highest matrix density with a well-ordered diamond–matrix interface. This microstructural integrity suppresses matrix wear, prevents the continuous exposure of fresh diamond grains, and consequently demands higher energy input during cutting—directly explaining the peak SCE (1.4826 J/mm^3^) and Pnet (720.85 W) values recorded at this addition ratio.

Although the undoped socket (0% B_4_C) also yielded a comparably high SCE value (1.4553 J/mm^3^), SEM analysis revealed that this is attributable to a distinct mechanism: despite exhibiting low porosity (3.48%) and a dense matrix, the undoped socket displayed an approximately 8 µm interfacial gap at the diamond–matrix boundary (Figure 4b), indicative of poor bonding quality. This poor wetting behaviour reduces diamond protrusion efficiency, necessitating higher energy input to sustain cutting progress despite the comparatively low mechanical hardness of the matrix.

The lowest SCE (0.9044 J/mm^3^) and Pnet (430.26 W) values were obtained for the 7% B_4_C-doped circular saw. SEM observations confirmed that at 7% B_4_C, pore sizes exceed 40 µm, accompanied by heterogeneous B_4_C distribution and weak interfacial bonding (Figure 4o,p). The resultant high porosity accelerates matrix wear, enabling continuous exposure of fresh diamond grains that facilitate cutting with minimal energy input. The progressive decline in both SCE and Pnet observed between 5% and 7% B_4_C doping is fully consistent with the sharp increase in porosity (8.70% → 11.93% → 14.31%) and the deteriorating matrix quality documented in both metallographic measurements and SEM analyses. The SCE and net power consumption values obtained from the cutting tests are presented in Table 4.

#### 3.3.2. Specific Wear

In natural stone cutting operations, the optimal wear of both the diamond and matrix is essential to maximise the service life of the cutting tool. Accordingly, the selection of an appropriate matrix composition and diamond type for the sockets is of critical importance [39].

The reduction in socket height recorded after the cutting tests was divided by the total cut area to determine specific wear (SW) values, expressed in µc/m^2^. The lowest SW value was obtained for the 4% B_4_C-doped circular saw, whose superior wear resistance is attributed to the suppression of matrix fragment detachment resulting from the higher hardness achieved at this addition ratio—as confirmed by SEM analysis. Conversely, the highest SW value was recorded for the 7% B_4_C-doped socket, which exhibited the highest porosity among all compositions tested. The specific wear values obtained from the cutting tests are presented in Table 5. Specific wear values are based on a single measurement per composition. Derived from the difference in socket height before and after 60 consecutive cuts, divided by the total cut area. As only one saw blade per composition was used in the cutting tests, no standard deviation could be calculated for this parameter.

#### 3.3.3. Relationship Between Net Power Consumption and Metallographic Properties

A linear increasing relationship between net power consumption and unit volume weight was found (r = 0.70, Figure 6). Net power consumption values increased with increasing socket density. This behavior is microstructurally supported by SEM observations: at higher UVW values (e.g., 4% B_4_C), the homogeneous B_4_C distribution and well-bonded matrix–diamond interface result in a harder, denser structure that resists wear and requires more energy for cutting.

A negative exponential relationship between net power consumption and porosity (r = 0.87, Figure 7) was determined. As porosity increases, socket wear increases, new diamond grains are continuously exposed to the socket surface, facilitating the cutting process and reducing net power consumption. SEM images are consistent with this mechanism: the large pores (35–40 µm) observed in 6% and 7% B_4_C sockets directly accelerate matrix wear and diamond exposure.

The primary regression analysis of net power consumption and Knoop hardness, based on the full dataset (n = 8), yielded a correlation coefficient of r = 0.56 (Figure 8). When the undoped socket (0% B_4_C) was excluded as a sensitivity analysis (n = 7), the correlation strengthened to r = 0.76 (Figure 9). This improvement can be attributed to the fundamentally different diamond–matrix bonding mechanism of the undoped socket (characterised by an interfacial gap of approximately 8 µm confirmed by SEM analysis), a feature absent in all B_4_C-doped compositions.

A negative linear relationship between net power consumption and abrasion loss by weight (r = 0.78, Figure 10) was determined. Power consumption decreased as socket wear increased. The 4% B_4_C socket showed the lowest wear and highest power consumption, consistent with SEM evidence of its dense, well-bonded, homogeneous microstructure.

The regression statistics, including sample size, regression equations, correlation coefficients, coefficients of determination, and *p*-values for all Pnet–metallographic property relationships, are summarised in Table 6.

#### 3.3.4. Relationships Between Specific Cutting Energy and Metallographic Properties

An increasing linear relationship between specific cutting energy and unit volume weight (r = 0.70, Figure 11) was obtained. A very high negative exponential correlation (r = 0.88) was obtained between SCE and porosity (Figure 12), suggesting porosity as the most influential metallographic predictor of specific cutting energy. SEM observations provide a plausible explanation: the progressive increase in pore size from ~2–4 µm (0% B_4_C) to >40 µm (7% B_4_C) observed in SEM images accelerates matrix wear, promotes diamond protrusion, and reduces the energy required for cutting.

The primary regression analysis of specific cutting energy and Knoop hardness, based on the full dataset (n = 8), yielded a correlation coefficient of r = 0.67 (Figure 13). As a sensitivity analysis, when the undoped socket (0% B_4_C) was excluded (n = 7), the correlation strengthened markedly to r = 0.92 (Figure 14). This contrast is explained by SEM: the undoped socket’s weak diamond–matrix bonding (~8 µm interface gap) causes a fundamentally different cutting behaviour compared to sockets with well-bonded interfaces achieved through B_4_C doping.

The highest hardness values are obtained depending on the matrix composition with a properly densified matrix–diamond mixture. As socket hardness increases, matrix wear decreases and the emergence of new diamond grains at the socket surface is suppressed; consequently, higher energy is required for cutting, leading to increased specific cutting energy values. Consistent with this mechanism, an exponentially decreasing relationship between SCE and abrasion loss by weight was obtained (r = 0.84, Figure 15). Sockets with harder and denser matrices—as supported by SEM observations—consume greater energy during cutting, whereas sockets exhibiting high abrasion loss display lower SCE values, consistent with accelerated matrix wear and continuous diamond exposure. Collectively, these findings demonstrate that both hardness and wear resistance are closely interrelated metallographic indicators of specific cutting energy, and the underlying mechanisms of both parameters are consistent with SEM microstructural observations.

The regression statistics, including sample size, regression equations, correlation coefficients, coefficients of determination, and *p*-values for all SCE–metallographic property relationships, are summarised in Table 7.

#### 3.3.5. Relationships Between Specific Wear and Metallographic Properties

The relationships between the unit volume weight, porosity, and hardness values of the sockets—together with the wear amount values determined from weight loss measurements—and the specific wear values obtained from the cutting tests were examined. High correlation coefficients were obtained between specific wear (SW) values and most of the metallographic properties investigated.

A decreasing linear relationship between specific wear and unit volume weight (r = 0.87, Figure 16) was determined. An increasing linear relationship between specific wear and porosity (r = 0.97, Figure 17) was obtained—the highest correlation coefficient in this study. SEM images fully support this finding: the large pores (35–40 µm in 6% B_4_C; >40 µm in 7% B_4_C) provide weak matrix support, accelerate matrix material removal, and thus increase specific wear.

The primary regression analysis of specific wear and Knoop hardness, based on the full dataset (n = 8), yielded an exponentially decreasing relationship with a correlation coefficient of r = 0.69 (Figure 18). As a sensitivity analysis, when the undoped socket (0% B_4_C) was excluded (n = 7), the correlation strengthened to r = 0.83 (Figure 19). The 4% B_4_C socket, with its homogeneous B_4_C particle distribution (SEM-confirmed) and maximum hardness (HK = 303.46), exhibited the lowest specific wear (84.84 µc/m^2^), confirming the hardness–wear relationship at the microstructural level.

A linear relationship with a very high correlation coefficient (r = 0.98, Figure 20) was found between specific wear and abrasion loss by weight, suggesting that specific wear of circular saws can be reliably estimated from laboratory weight loss tests before cutting operations.

The regression statistics, including sample size, regression equations, correlation coefficients, coefficients of determination, and *p*-values for all SW–metallographic property relationships, are summarised in Table 8.

## 4. Conclusions

In this study, the relationships between the metallographic properties of B_4_C-doped diamond sockets and granite machinability parameters were examined through simple regression analysis, and SEM-based microstructural observations were used to interpret the identified relationships. The findings are as follows:

1. Porosity was the most effective metallographic property for predicting machinability parameters, yielding the highest correlation coefficients with net power consumption (r = 0.87), specific cutting energy (r = 0.88), and specific wear (r = 0.97). SEM analyses revealed that pore size increased from 2–4 µm at 0% B_4_C to above 40 µm at 7% B_4_C, providing a direct microstructural basis for these relationships.
2. Among the compositions tested, the most homogeneous particle distribution and the strongest diamond–matrix interfacial bond were obtained at the 4% B_4_C composition. However, this composition also resulted in the highest net power consumption, indicating a performance trade-off rather than an unequivocal optimum.
3. Despite exhibiting moderate metallographic values, the undoped socket (0% B_4_C) displayed a diamond–matrix interfacial gap of approximately 8 µm in SEM images, which is indicative of poor bonding quality. This observation is consistent with the lower regression coefficients obtained in analyses that included this composition in the dataset.
4. Above 5% B_4_C, SEM images revealed non-homogeneous particle distribution, coarse matrix grains, and weak interfacial bonding. These findings are consistent with the sharp increases in porosity and specific wear recorded at these addition levels. Accordingly, optimization of sintering parameters for higher B_4_C contents is recommended in future studies.

The regression models developed in this study demonstrate that metallographic properties are meaningful indicators of cutting performance trends and may assist in the preliminary evaluation of socket compositions. However, independent validation under different rock types and cutting conditions is required before broader generalizations can be drawn.

## Figures and Tables

**Figure 1:**
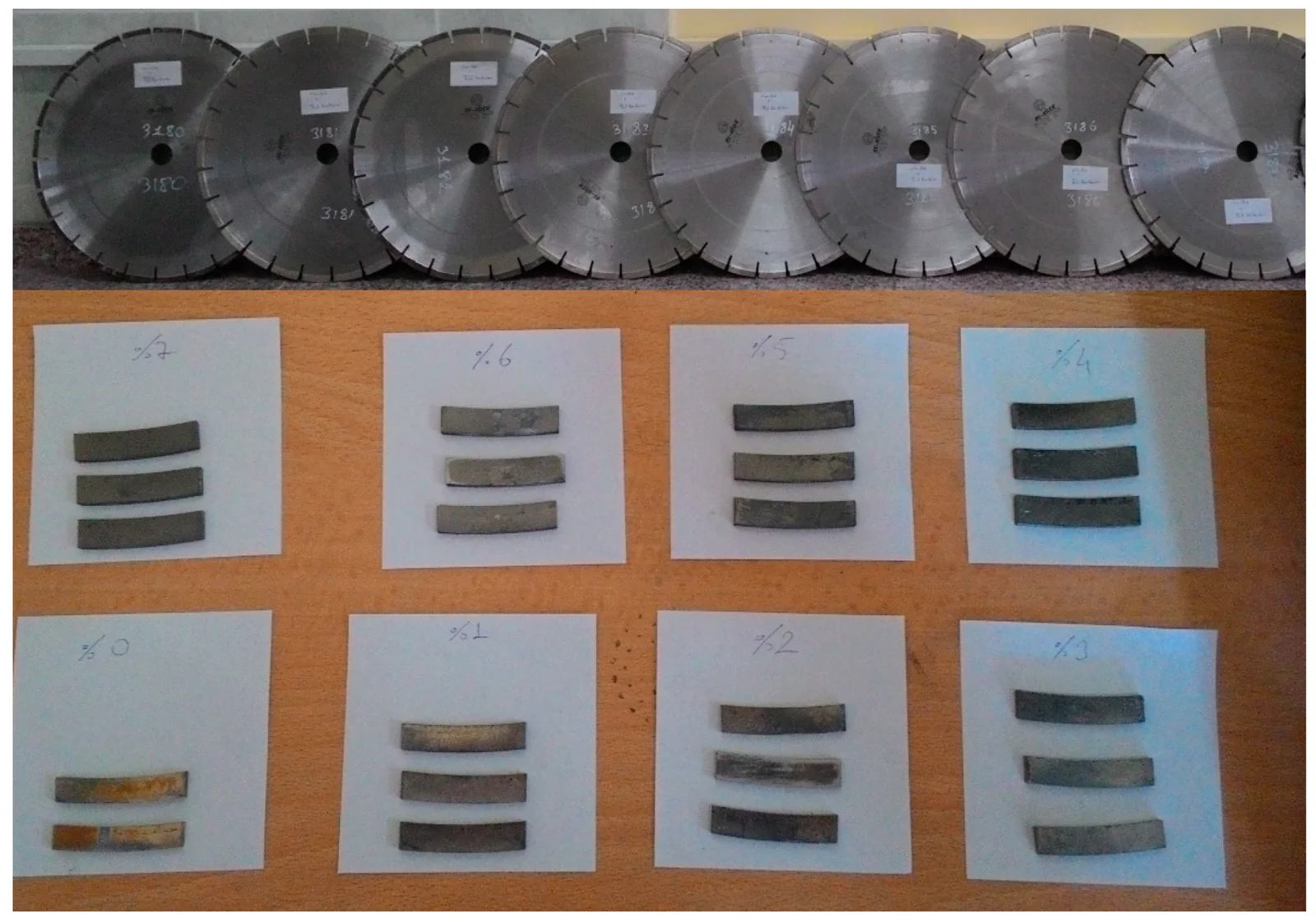
General views of the produced B_4_C-doped diamond sockets and the circular saws used in granite cutting experiments.

**Figure 2:**
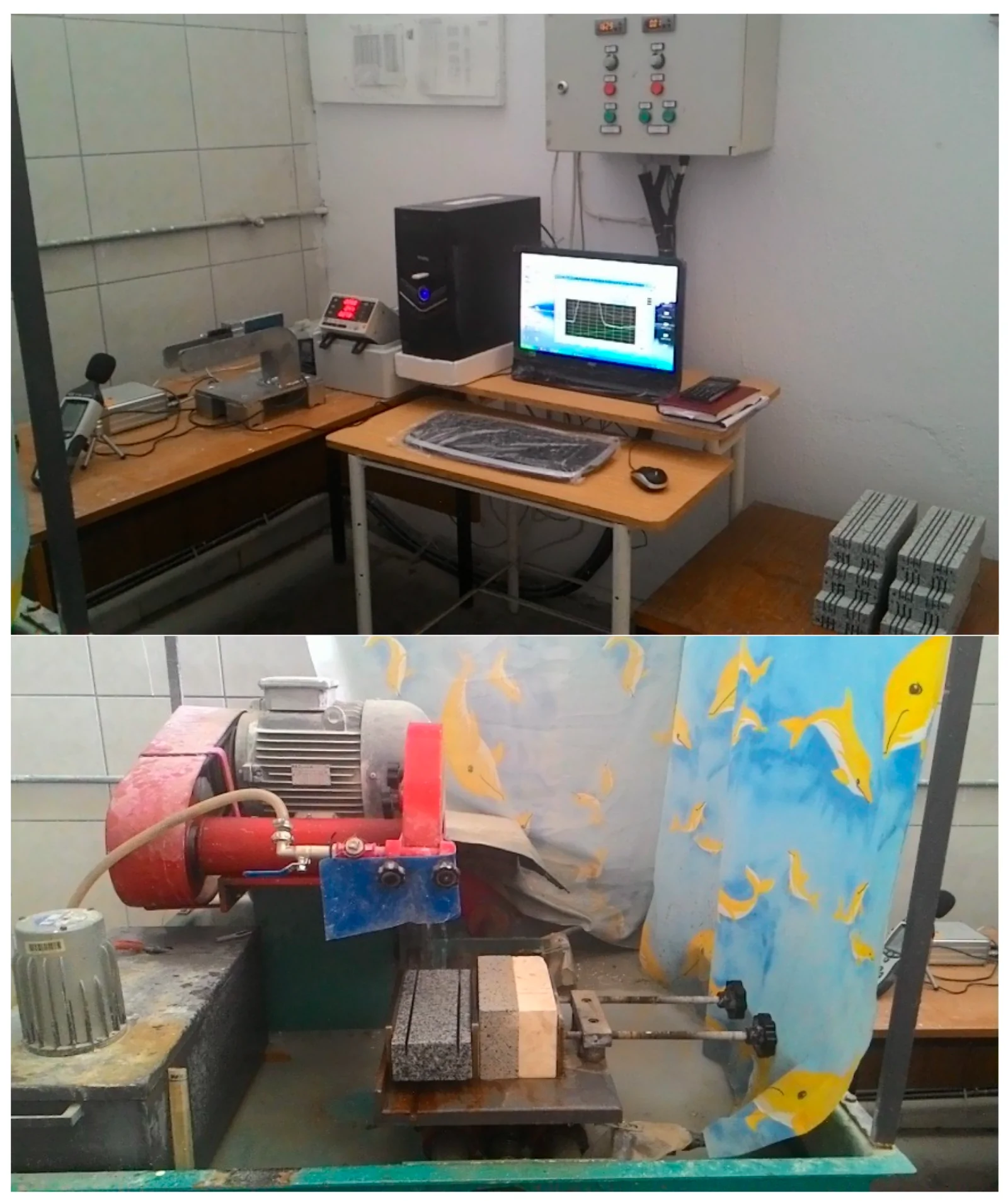
General view of the cutting test set-up and view during cutting.

**Figure 3:**
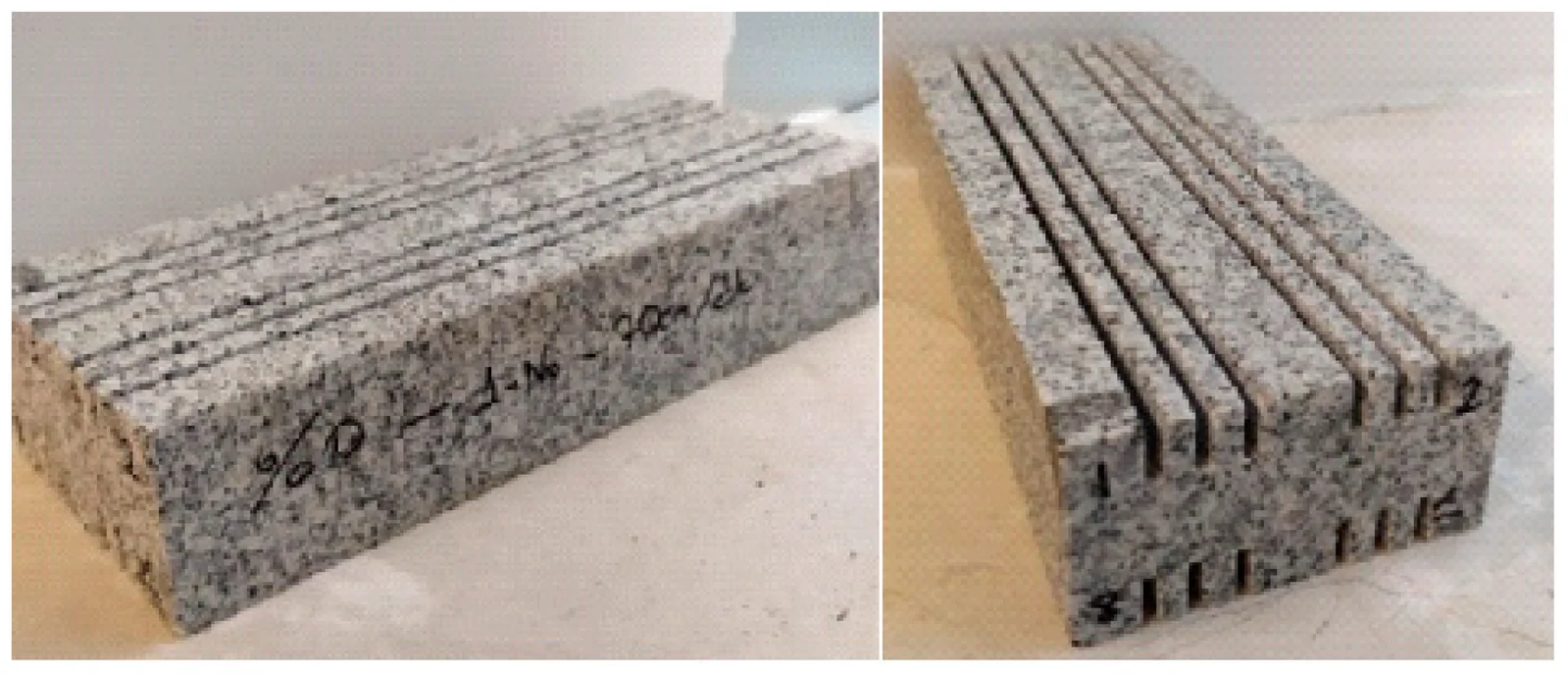
View of specimens after cutting tests.

**Figure 4:**
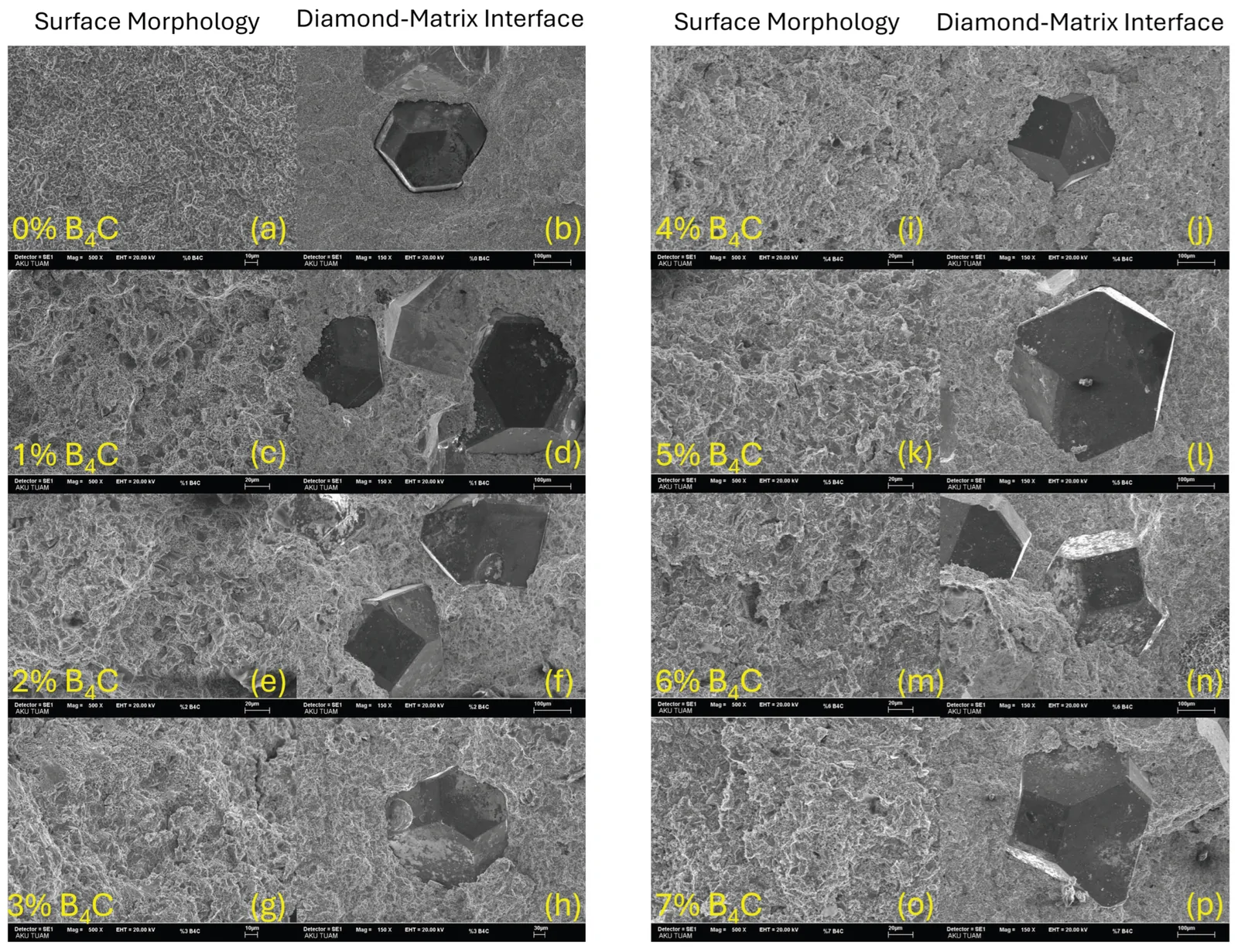
(**a**–**p**) SEM images of sockets with varying B_4_C addition ratios: (**a**,**b**) 0% B_4_C, (**c**,**d**) 1% B_4_C, (**e**,**f**) 2% B_4_C, (**g**,**h**) 3% B_4_C, (**i**,**j**) 4% B_4_C, (**k**,**l**) 5% B_4_C, (**m**,**n**) 6% B_4_C, (**o**,**p**) 7% B_4_C [41].

**Figure 5:**
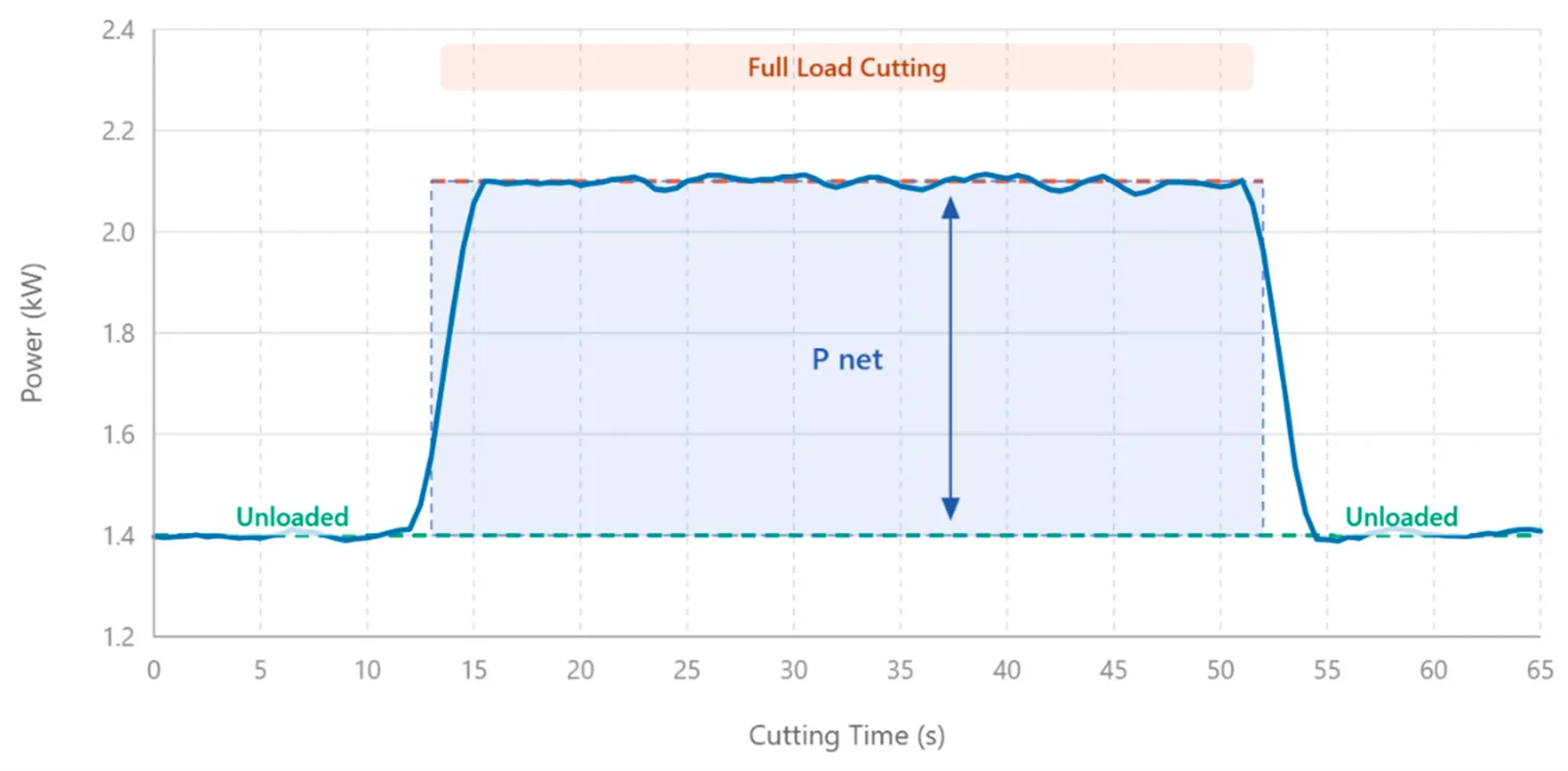
Power–time graph during cutting SCE values were calculated using Equation (5) [25].

**Figure 6:**
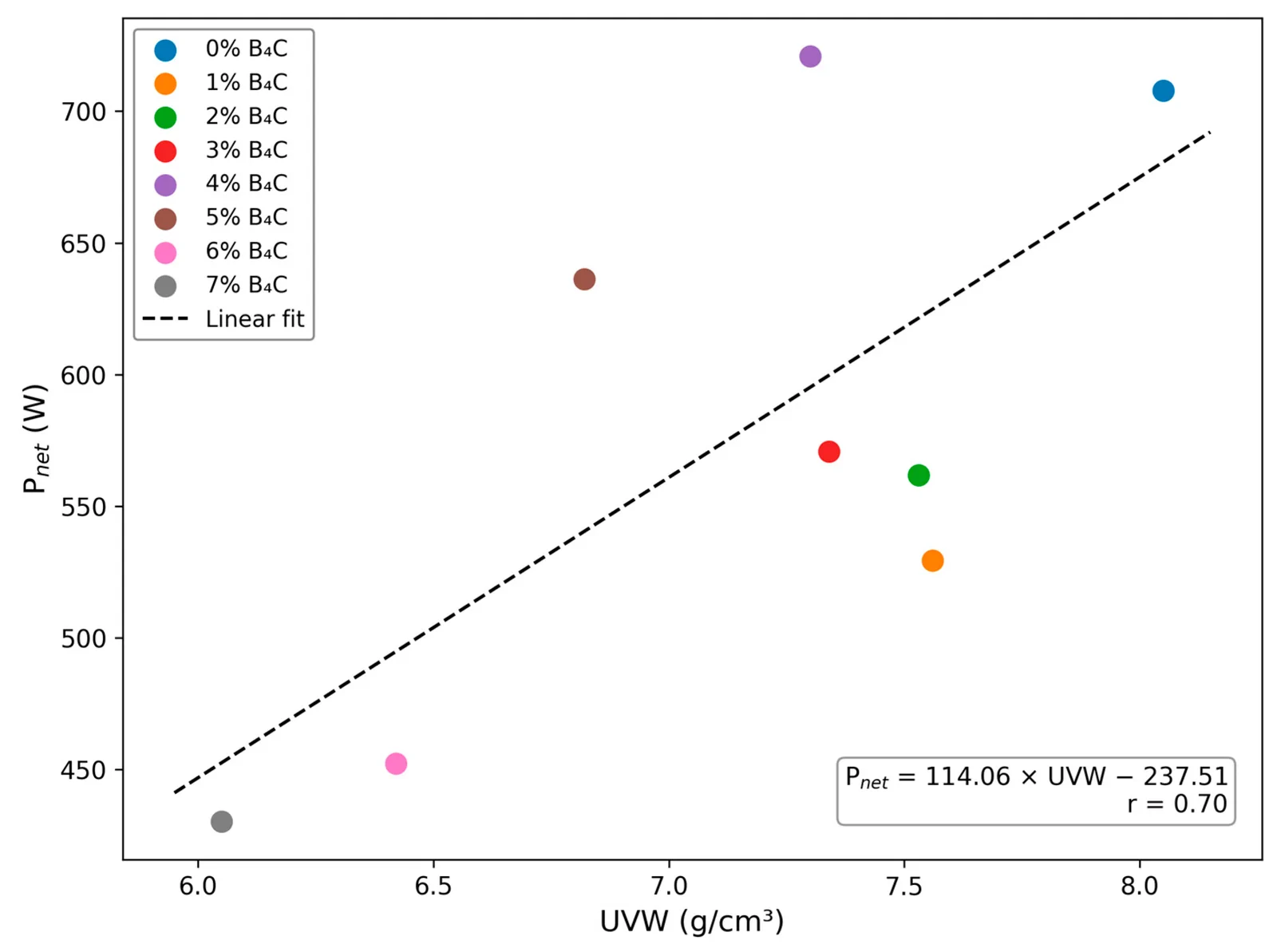
Relationship between net power consumption (Pnet) and unit volume weight (UVW).

**Figure 7:**
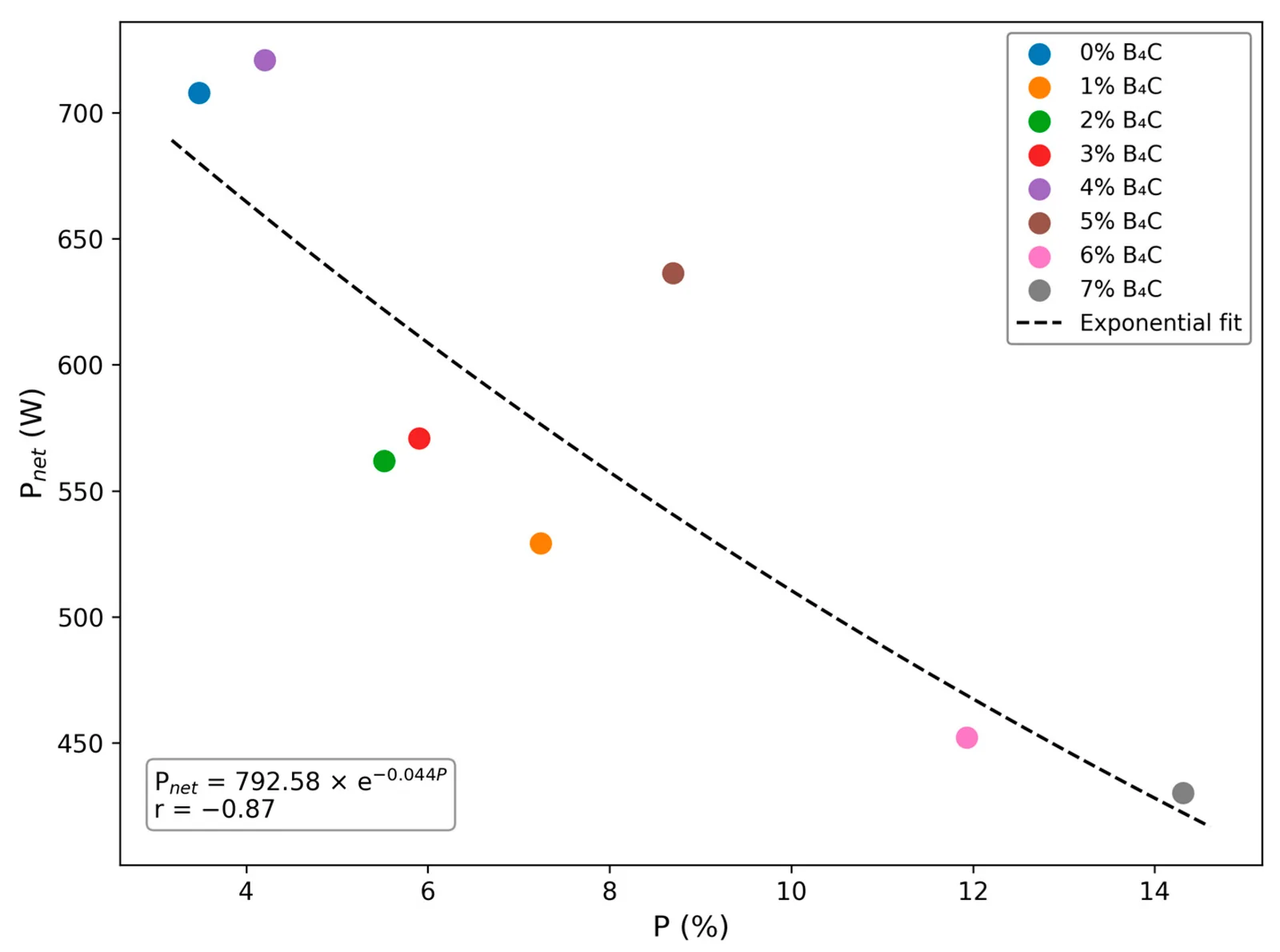
Relationship between net power consumption (Pnet) and porosity (P).

**Figure 8:**
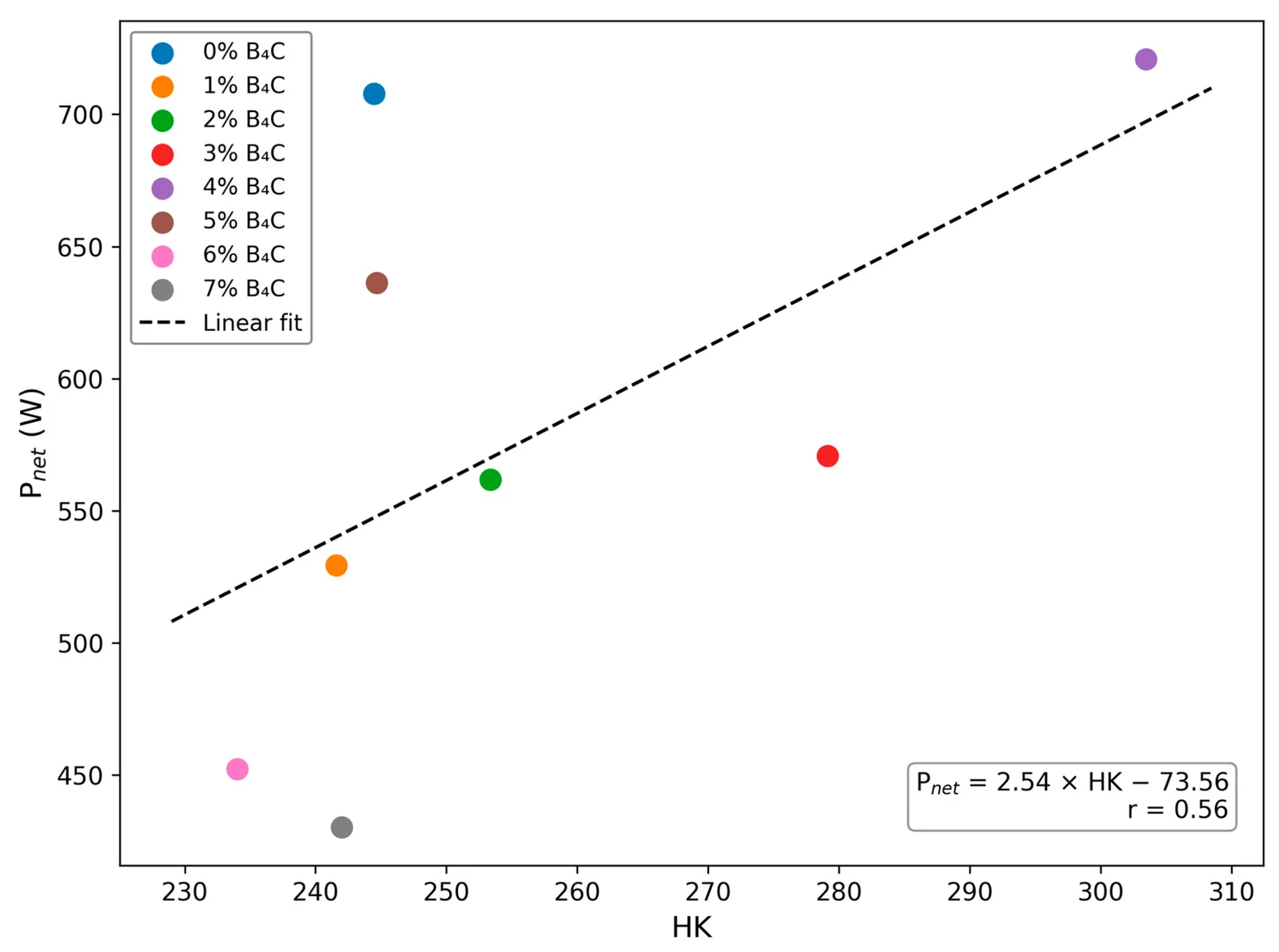
Relationship between net power consumption (Pnet) and Knoop hardness (HK) (including 0% B_4_C).

**Figure 9:**
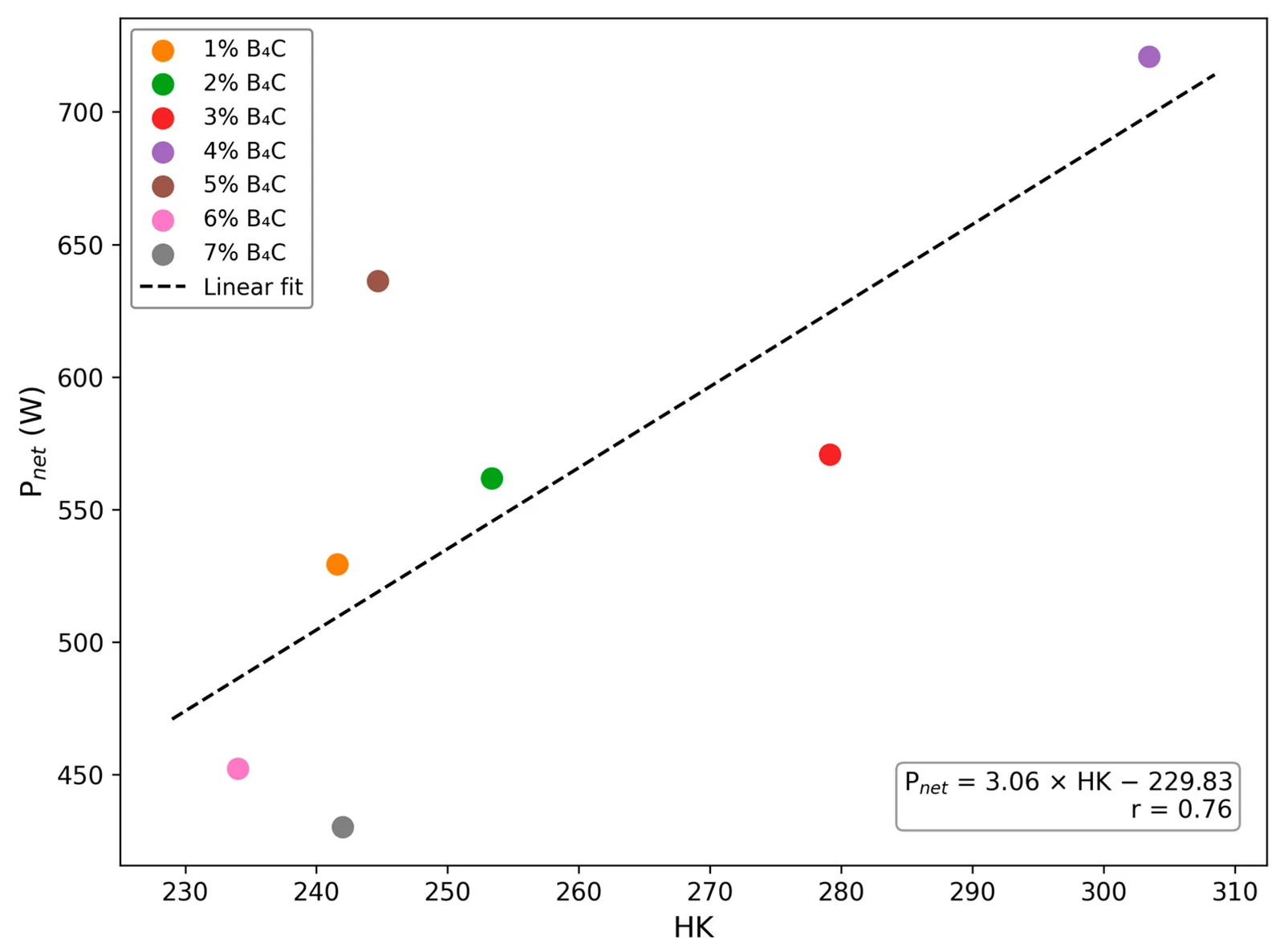
Relationship between net power consumption (Pnet) and Knoop hardness (HK) (excluding 0% B_4_C).

**Figure 10:**
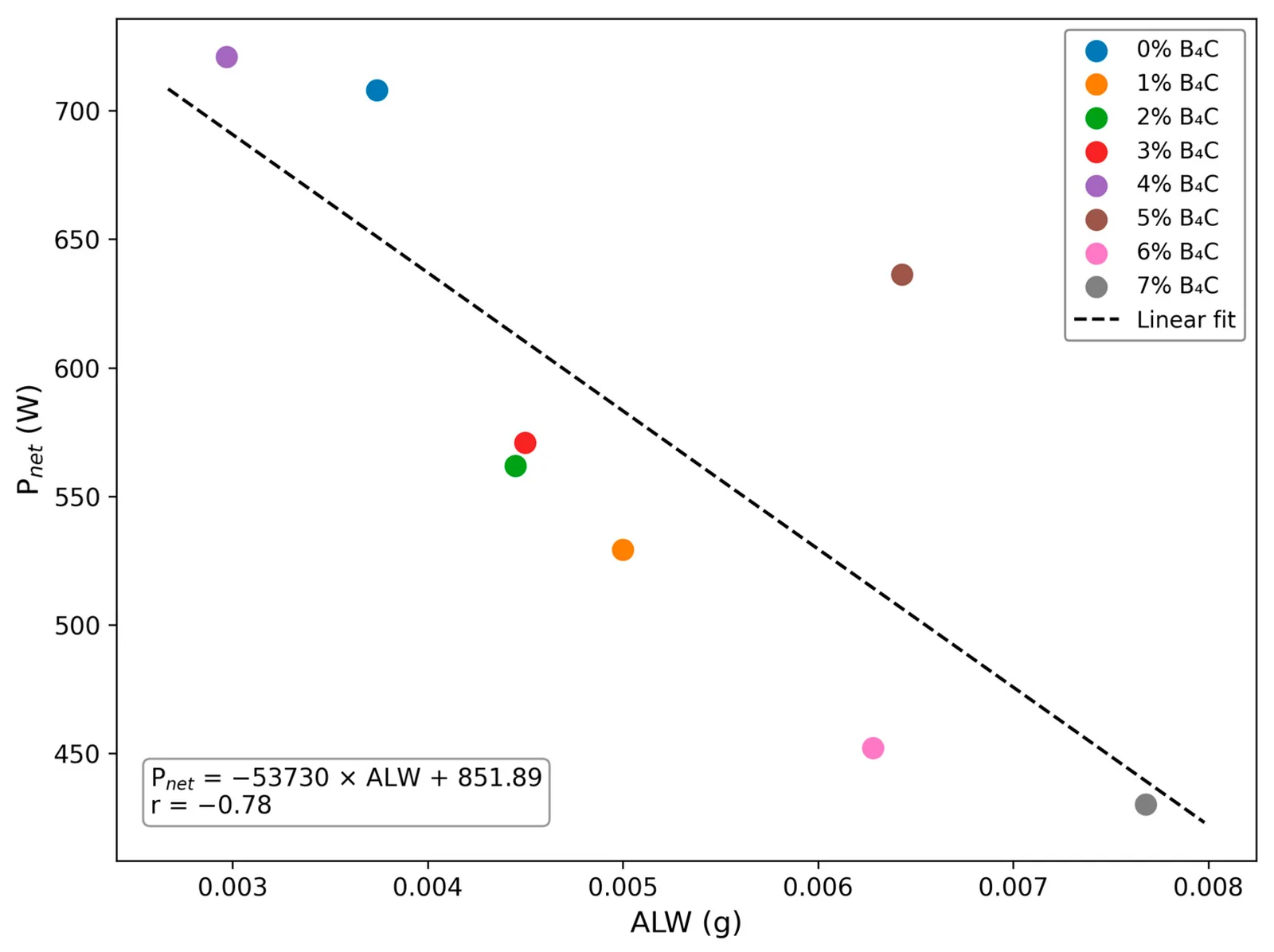
Relationship between net power consumption (Pnet) and abrasion loss by weight (ALW).

**Figure 11:**
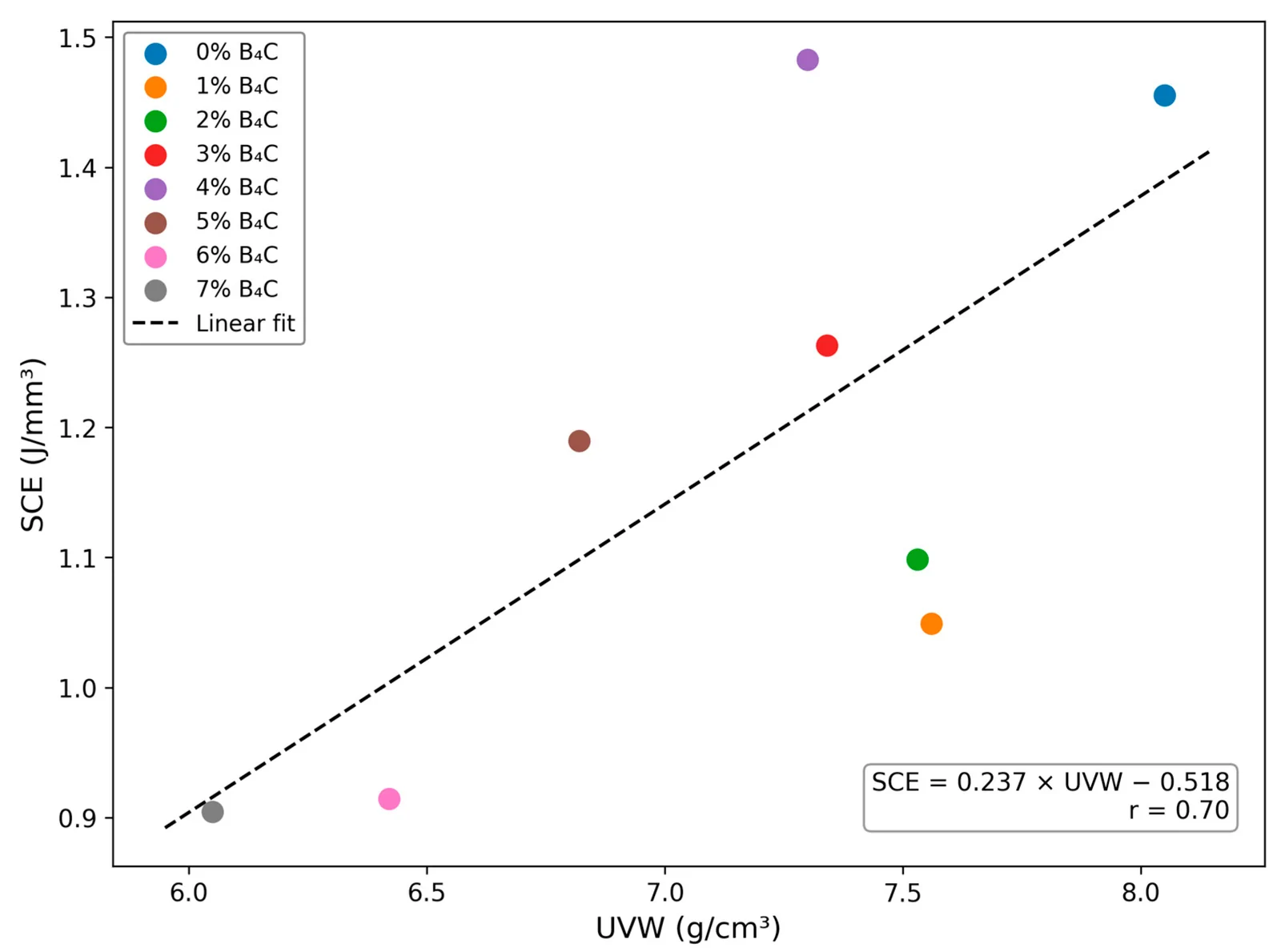
Relationship between specific cutting energy and unit volume weight.

**Figure 12:**
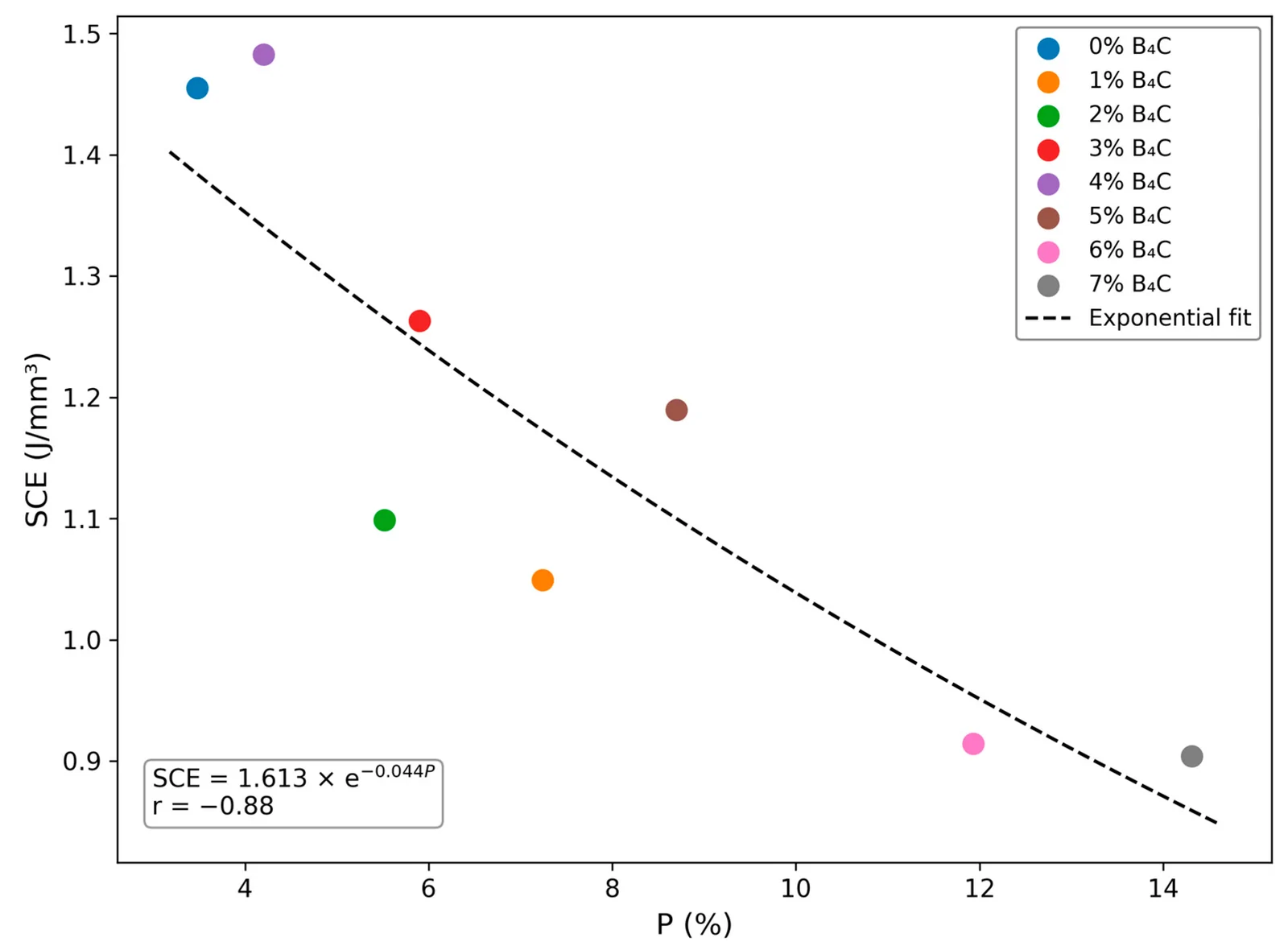
Relationship between specific cutting energy (SCE) and porosity (P).

**Figure 13:**
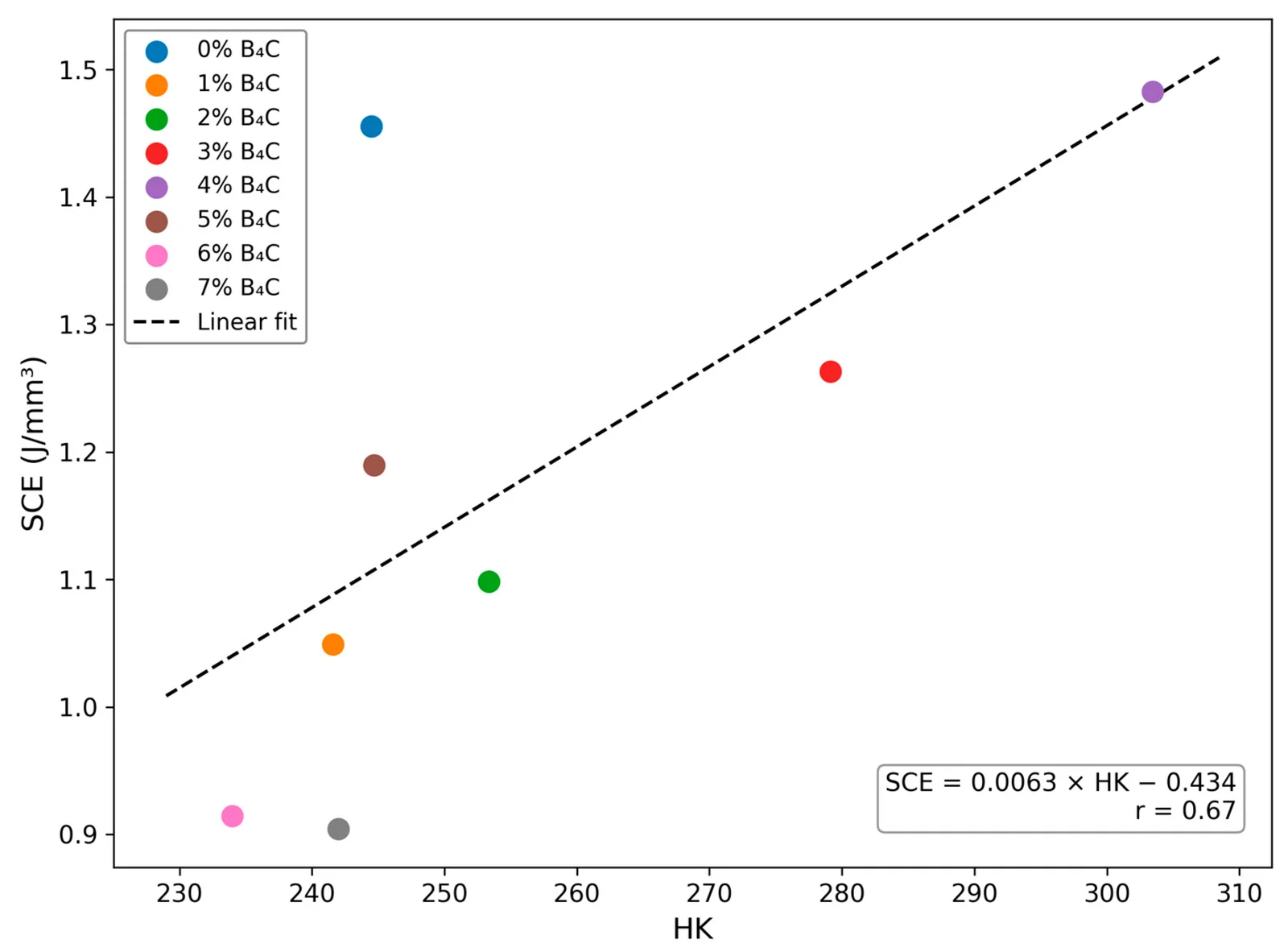
Relationship between specific cutting energy (SCE) and Knoop hardness (HK) (including 0% B_4_C).

**Figure 14:**
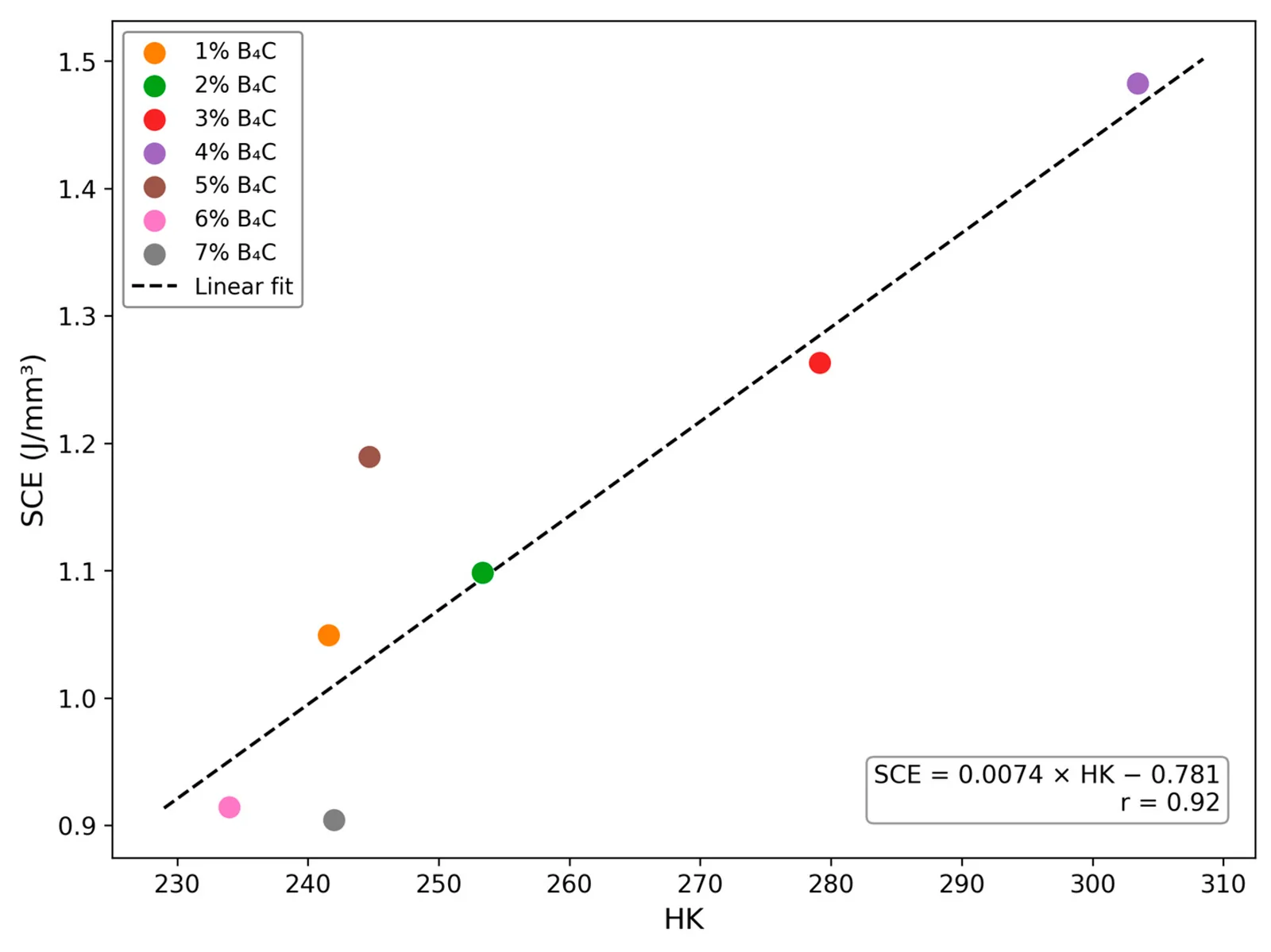
Relationship between specific cutting energy (SCE) and Knoop hardness (HK) (excluding 0% B_4_C).

**Figure 15:**
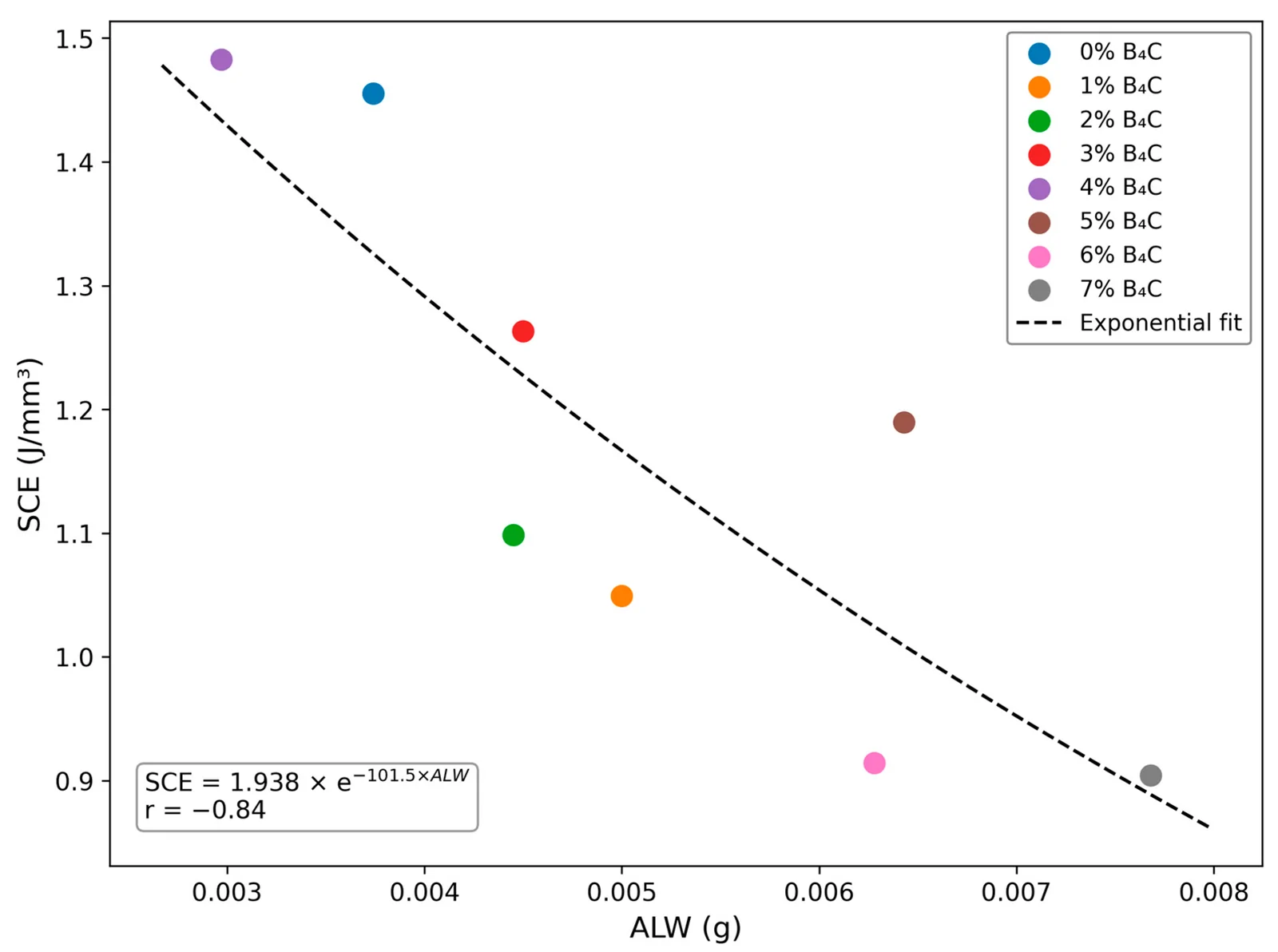
Relationship between specific cutting energy (SCE) and abrasion loss by weight (ALW).

**Figure 16:**
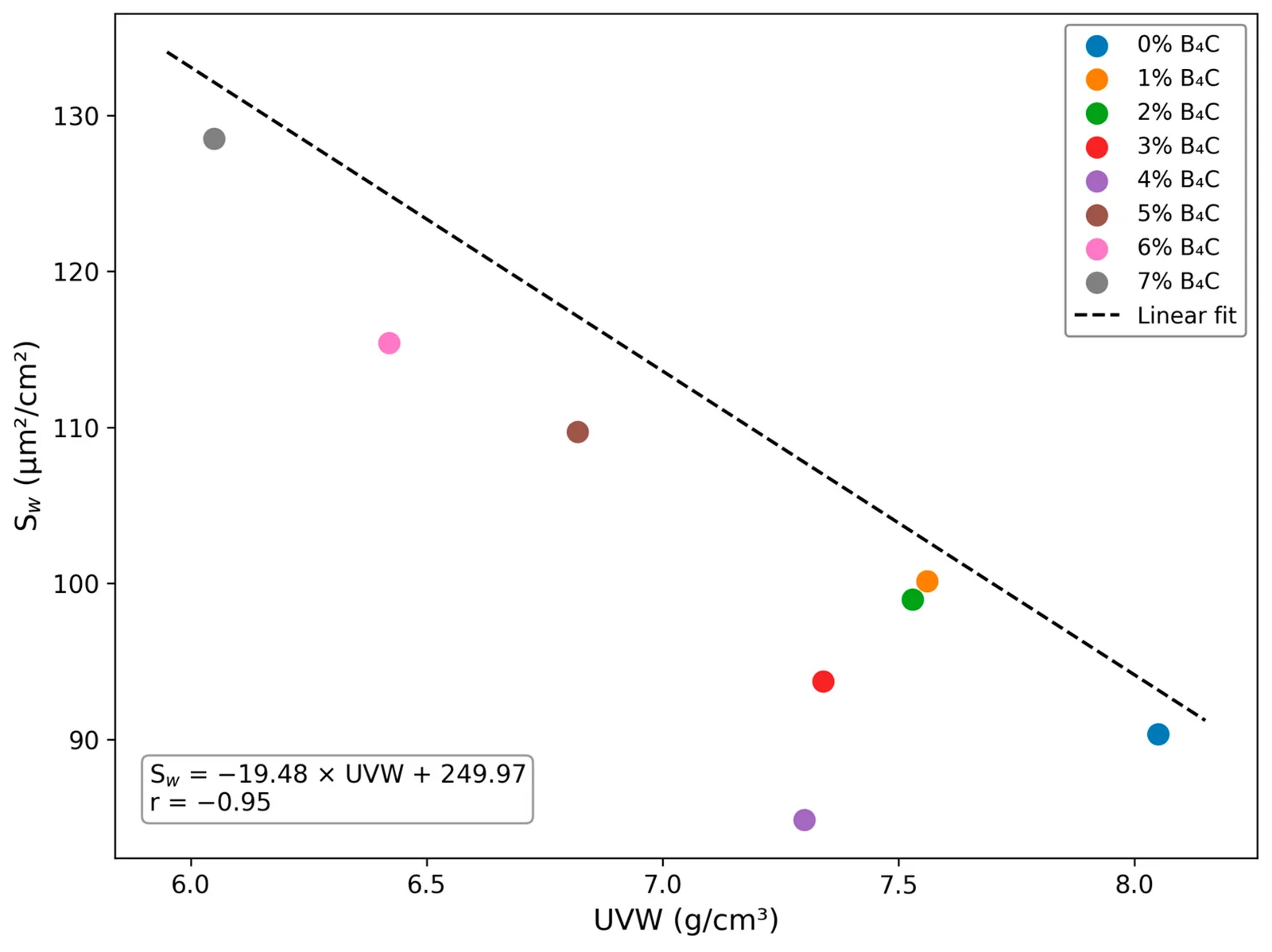
Relationship between specific wear (SW) and unit volume weight (UVW).

**Figure 17:**
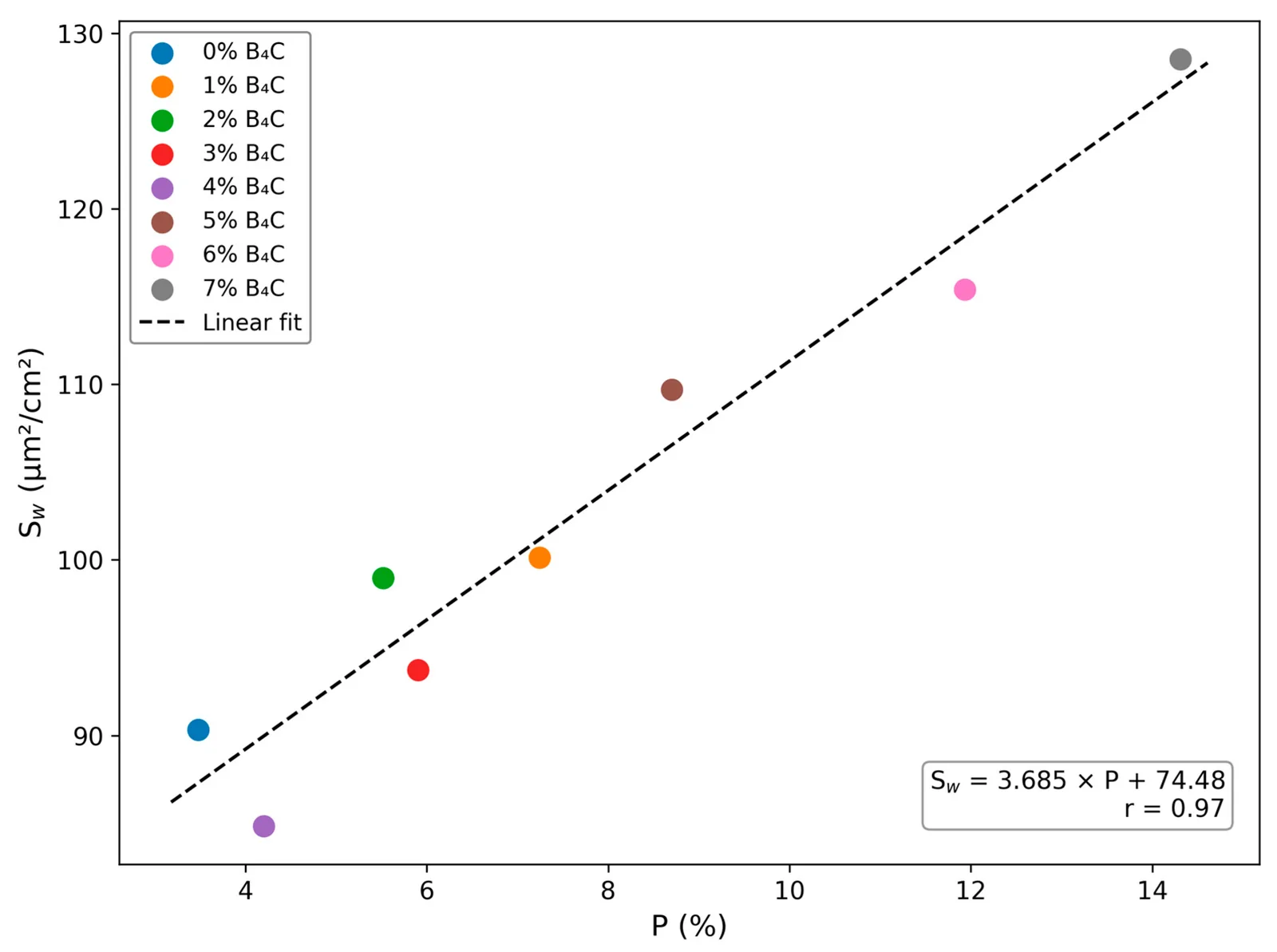
Relationship between specific wear (SW) and porosity (P).

**Figure 18:**
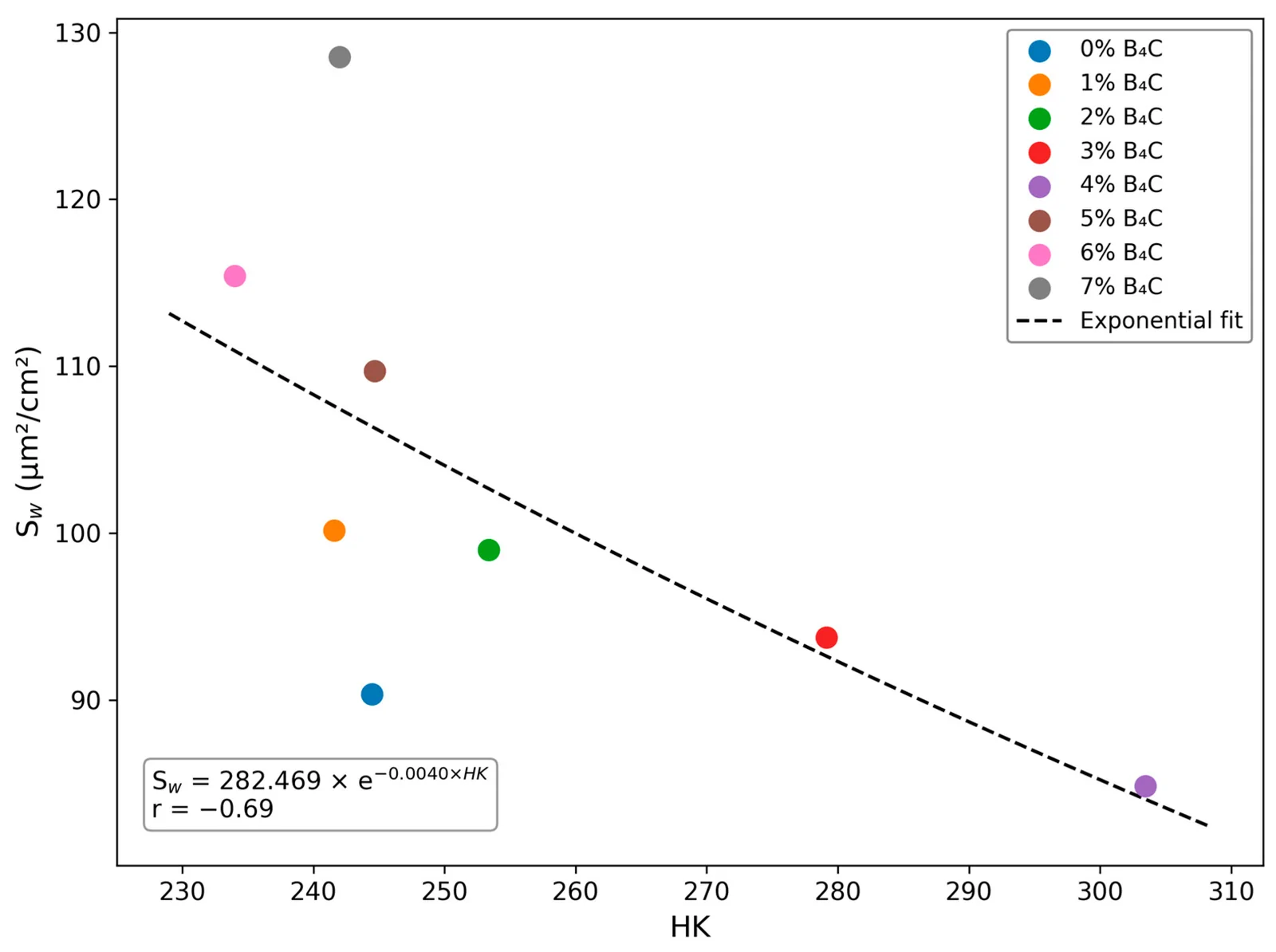
Relationship between specific wear (SW) and Knoop hardness (HK) (including 0% B_4_C).

**Figure 19:**
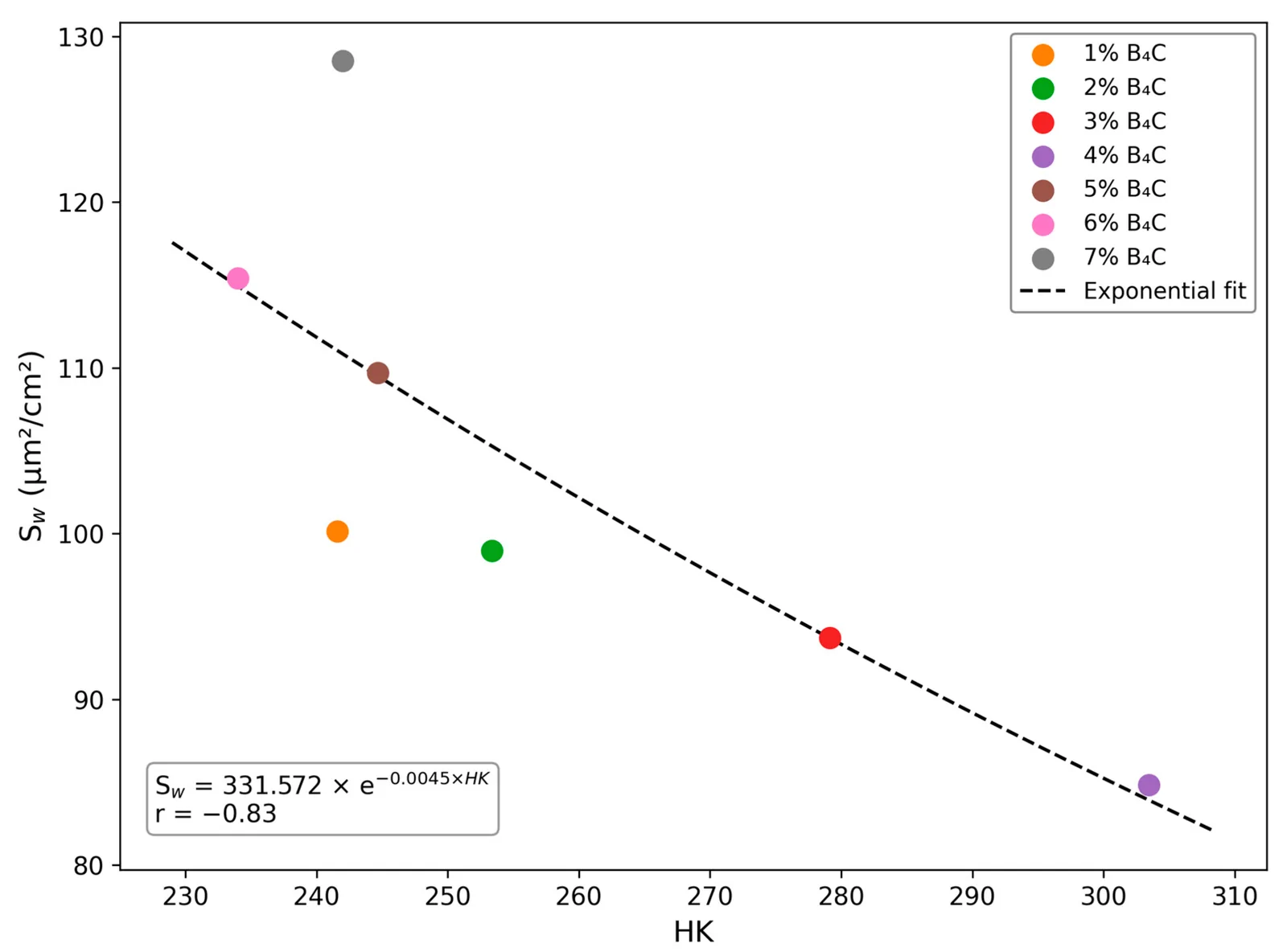
Relationship between specific wear (SW) and Knoop hardness (HK) (excluding 0% B_4_C).

**Figure 20:**
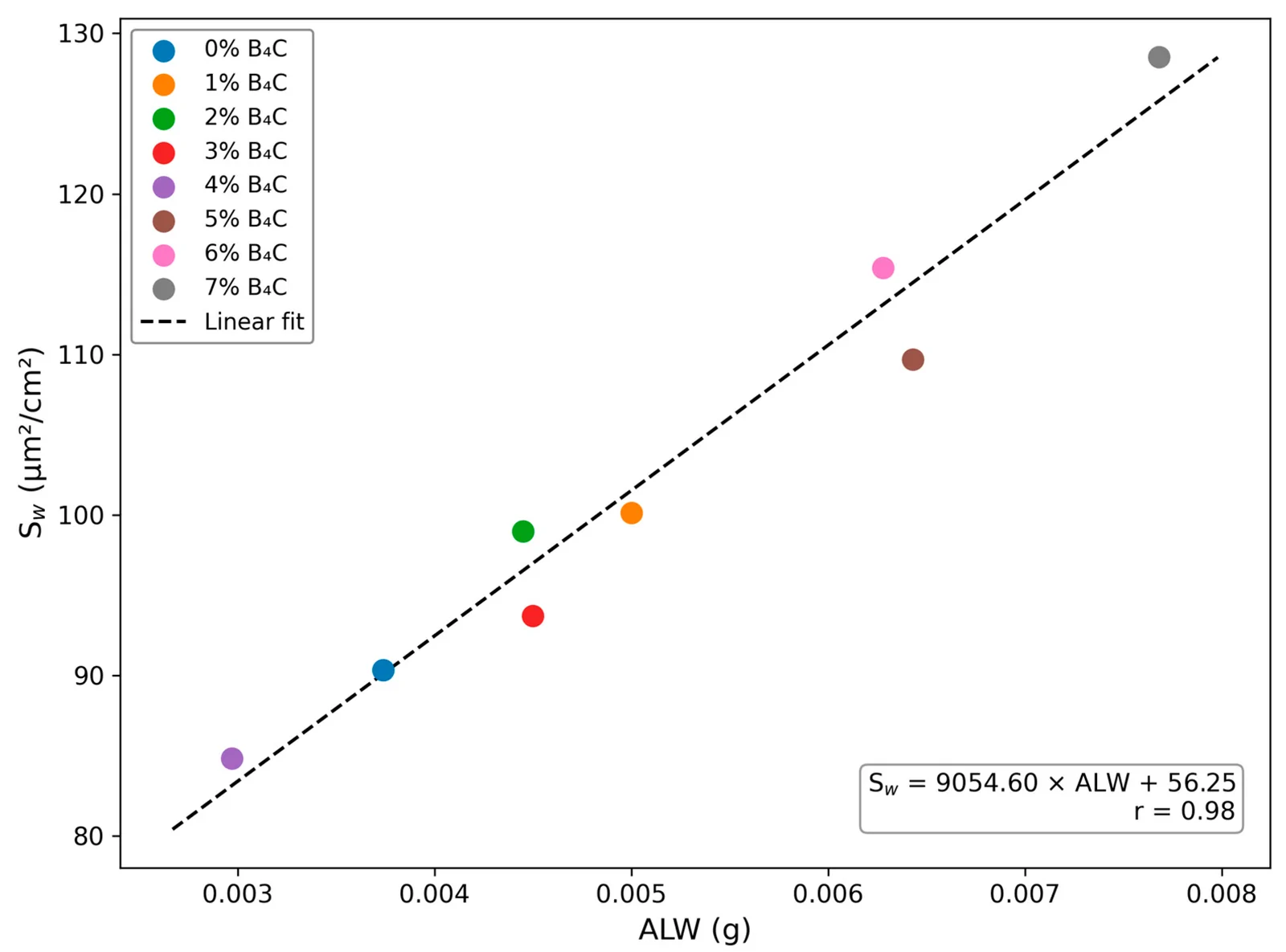
Relationship between specific wear (SW) and abrasion loss by weight (ALW).

**Table 1:** Matrix compositions of the B_4_C-doped diamond sockets.

B_4_C (wt.%)	Cobalt (wt.%)	Bronze (wt.%)	Nickel (wt.%)
0	70	29	1
1	69	29	1
2	68	29	1
3	67	29	1
4	66	29	1
5	65	29	1
6	64	29	1
7	63	29	1

**Table 2:** Cutting conditions maintained constant throughout the study [41].

Peripheral Speed (Vp) m/s	Depth of Cut (Dc), mm	Feed Rate (Vi), m/min
30	10	0.7

**Table 3:** Metallographic properties of diamond sockets [41].

B_4_C (wt.%)	UVW (g/cm^3^)		P (%)		HK		ALW (g)		No. of Experiments
	Mean	Standard Deviation	Mean	Standard Deviation	Mean	Standard Deviation	Mean	Standard Deviation	
0	8.05	0.11	3.48	0.68	244.47	49.79	0.00374	0.0012	3
1	7.56	0.15	7.24	1.81	241.58	59.13	0.00500	0.0004	3
2	7.53	0.12	5.52	1.48	253.35	53.41	0.00445	0.0045	3
3	7.34	0.04	5.90	0.48	279.13	60.66	0.00450	0.0017	3
4	7.30	0.02	4.20	1.88	303.46	65.94	0.00297	0.0019	3
5	6.82	0.07	8.70	0.91	244.67	60.04	0.00643	0.0021	3
6	6.42	0.06	11.93	0.75	234	53.56	0.00628	0.0063	3
7	6.05	0.11	14.31	0.48	242	63.66	0.00768	0.0032	3

*UVW: unit volume weight, P: porosity (P), HK: Knoop hardness, ALW: abrasion loss by weight.*

**Table 4:** SCE and Pnet consumption values [41].

B_4_C (wt.%)	SCE (J/mm^3^)		Pnet (watt)		Number of Cuts
	Mean	Standard Deviation	Mean	Standard Deviation	
0	1.4553	0.051	707.85	28.87	60
1	1.0493	0.095	529.32	57.14	60
2	1.0986	0.081	561.86	50.09	60
3	1.2632	0.069	570.81	53.60	60
4	1.4826	0.076	720.85	48.25	60
5	1.1896	0.078	636.28	37.05	60
6	0.9144	0.075	452.22	36.55	60
7	0.9044	0.061	430.26	33.52	60

SCE: specific cutting energy, Pnet: net power consumption.

**Table 5:** Specific wear (SW) values [41].

B_4_C (wt.%)	Specific Wear (SW) (µc/m^2^)
0	90.34
1	100.15
2	98.98
3	93.73
4	84.84
5	109.70
6	115.41
7	128.52

**Table 6:** Regression statistics for the relationships between net power consumption (Pnet) and metallographic properties.

Independent Variable	n	Model	Equation	r	R^2^	*p*
UVW	8	Linear	Pnet = 114.06 × UVW − 237.51	0.70	0.487	0.054
P	8	Exponential	Pnet = 792.58 × e^(−0.044P)	−0.87	0.7351	0.005
HK	8	Linear	Pnet = 2.54 × HK − 73.56	0.56	0.3151	0.148
HK *	7	Linear	Pnet = 3.06 × HK − 229.83	0.76	0.5843	0.045
ALW	8	Linear	Pnet = −53730 × ALW + 851.89	−0.78	0.6058	0.02

* UVW: unit volume weight, P: porosity (P), HK: Knoop hardness, ALW: abrasion loss by weight.

**Table 7:** Regression statistics for the relationships between specific cutting energy (SCE) and metallographic properties.

Independent Variable	n	Model	Equation	r	R^2^	*p*
UVW	8	Linear	SCE = 0.237 × UVW − 0.518	0.70	0.493	0.052
P	8	Exponential	SCE = 1.613 × e^(−0.044 × P)	−0.88	0.7692	0.004
HK	8	Linear	SCE = 0.0063 × HK − 0.434	0.67	0.452	0.068
HK *	7	Linear	SCE = 0.0074 × HK − 0.781	0.92	0.840	0.004
ALW	8	Exponential	SCE = 1.938 × e^(−101.5 × ALW)	−0.84	0.7186	0.010

* UVW: unit volume weight, P: porosity (P), HK: Knoop hardness, ALW: abrasion loss by weight.

**Table 8:** Regression statistics for the relationships between specific wear (SW) and metallographic properties.

Independent Variable	n	Model	Equation	r	R^2^	*p*
UVW	8	Linear	SW = −19.12 × UVW + 239.12	−0.87	0.764	0.005
P	8	Linear	SW = 3.685 × P + 74.48	0.97	0.944	<0.001
HK	8	Exponential	SW = 282.47 × e^(−0.004 × HK)	−0.69	0.447	0.057
HK *	7	Exponential	SW = 331.57 × e^(−0.0045 × HK)	−0.83	0.631	0.022
ALW	8	Linear	SW = 9054.6 × ALW + 56.25	0.98	0.961	<0.001

* UVW: unit volume weight, P: porosity (P), HK: Knoop hardness, ALW: abrasion loss by weight.

## Data Availability

The original contributions presented in this study are included in the article. Further inquiries can be directed to the corresponding author.
